# Pareto Optimality Explanation of the Glycolytic Alternatives in Nature

**DOI:** 10.1038/s41598-019-38836-9

**Published:** 2019-02-22

**Authors:** Chiam Yu Ng, Lin Wang, Anupam Chowdhury, Costas D. Maranas

**Affiliations:** Department of Chemical Engineering, The Pennsylvania State University, University Park, PA 16802 USA

## Abstract

The Entner-Doudoroff (ED) and Embden-Meyerhof-Parnas (EMP) glycolytic pathways are largely conserved across glycolytic species in nature. Is this a coincidence, convergent evolution or there exists a driving force towards either of the two pathway designs? We addressed this question by first employing a variant of the optStoic algorithm to exhaustively identify over 11,916 possible routes between glucose and pyruvate at different pre-determined stoichiometric yields of ATP. Subsequently, we analyzed the thermodynamic feasibility of all the pathways at physiological metabolite concentrations and quantified the protein cost of the feasible solutions. Pareto optimality analysis between energy efficiency and protein cost reveals that the naturally evolved ED and EMP pathways are indeed among the most protein cost-efficient pathways in their respective ATP yield categories and remain thermodynamically feasible across a wide range of ATP/ADP ratios and pathway intermediate metabolite concentration ranges. In contrast, pathways with higher ATP yield (>2) while feasible, are bound within stringent and often extreme operability ranges of cofactor and intermediate metabolite concentrations. The preponderance of EMP and ED is thus consistent with not only optimally balancing energy yield vs. enzyme cost but also with ensuring operability for wide metabolite concentration ranges and ATP/ADP ratios.

## Introduction

Billions of years of evolution led to highly genetically and phenotypically diverse organisms, yet most of them retain largely identical routes for sugar catabolism despite the presence of a myriad of ways in nature’s enzymatic repertoire for converting glucose to pyruvate^1^. Uniquely among them, the canonical Entner-Doudoroff (ED) and Embden-Meyerhof-Parnas (EMP) pathways are by far the most prevalent in nature^1^. These two pathways differ in the first few but share six of the remaining enzymatic steps. They both generate two moles of reduced redox cofactor NAD(P)H, which can be used for generating additional ATP through oxidative phosphorylation, but differ in the overall ATP yield. The ED pathway, often found in organisms living in carbon/energy/oxygen-rich environments (e.g., *Zymomonas mobilis*, *Acinetobacter sp*. *ADP1)*, sacrifices energy yield for a pathway with a larger driving force^1^. In addition, the canonical ED pathway confers higher tolerance to oxidative stress as it generates NADPH as opposed to NADH in the EMP pathway^2,3^. In contrast, the EMP pathway is common in prokaryotes and eukaryotes with higher energy demands or those living in anoxic or low-energy environments^1^. The presence of the key EMP enzyme 6-phosphofructokinase (PFK) or key ED enzymes 2-keto-3-deoxygluconate-6-phosphate (KDPG) aldolase and 6-phosphogluconate dehydratase is often used to identify whether a strain is capable of using either pathways^1,4^. The two pathways, however, are not mutually exclusive and often co-exist in many organisms^4,5^. In particular, enteric bacteria such as *Escherichia coli* can switch between them in response to the availability of different substrates^5,6^.

Variants of the canonical ED and EMP pathways have also been discovered especially in extremophiles^7^. Semi-phosphorylative and non-phosphorylative ED pathways were reported in anaerobic *Clostridia* and archaea, wherein the first ATP phosphorylation step is catalyzed by 2-dehydro-3-deoxy-D-gluconate (KDG) kinase or glycerate kinase, yielding one or zero ATP per glucose, respectively^8,9^. Modified EMP pathways are found in (hyper)thermophilic archaea employing variants of glycolytic enzymes utilizing alternative cofactors such as ADP-dependent glucokinase and PFK (in euryarchaeota), pyrophosphate (PP_i_)-dependent PFK (in *Thermoproteus tenax*)^10^, non-phosphorylating glyceraldehyde-3-phosphate (GAP) dehydrogenase (in crenarchaeon *Aeropyrum pernix* and *T*. *tenax*) and GAP ferredoxin oxidoreductase (in microaerobe *Pyrobaculum aerophilum*)^8,11,12^. A recent study confirmed that *Clostridium thermocellum* operates GTP and PP_i_-dependent glycolysis and predominantly employs a malate shunt to convert phosphoenolpyruvate (PEP) to pyruvate in the absence of pyruvate kinase^13^. An additional ATP per glucose by using a PP_i_-dependent PFK^9^ could potentially be gained, however, the source of PP_i_ in *C*. *thermocellum* remains elusive^13^. Another recently found variant of EMP relies on an NADP-dependent GAP dehydrogenase (in *Kluyveromyces lactis*)^14^. Notably, the NADP-dependent *C*. *acetobutylicum gapC* was expressed heterologously in *E*. *coli* replacing the native NAD-dependent GAP dehydrogenase to improve NADPH availability^15^. Alternate glucose processing pathways not conforming to the ED or EMP structure include coupling phosphoketolase (PK) pathway with the EMP pathway as a shunt for glycolysis by the heterofermentative lactic acid bacterium *Lactobacillus reuteri* ATCC 55730^16^. Bifidobacteria also utilize the bifid shunt for conversion of glucose to acetate: lactate: ATP in a 1.5:1:2.5 ratio^17^. In a groundbreaking work^18^, a non-oxidative glycolysis (NOG) was designed and constructed which operates cyclically to convert glucose to acetyl-coA in a redox-independent and carbon-neutral manner. The NOG pathway generates two ATPs and three acetate moieties per glucose molecule.

Previous studies have already tried to shed light on why the canonical glycolytic pathways are so uniquely prevalent despite the presence of many alternative routes with higher carbon and energy yields. A theoretical study by Melendez-Hevia *et al*. suggested that the canonical EMP pathway is an optimal series of chemical reactions that maximize ATP yield at high kinetic efficiency^19^. By designing a series of shortest pathways connecting different pairs of central carbon metabolites using a set of 30 reaction rules (that acts on carbohydrates), Noor *et al*. proposed that the canonical glycolytic pathway is the shortest pathway (in *E*. *coli*) that ensures the production of essential precursors of cellular biomass^20^. A recent biochemical analysis suggested that the glycolytic pathway uniquely manages to avoid toxic intermediates (e.g., methylglyoxal) and phosphorylates intermediates thus reducing metabolite leakage^21^. Furthermore, a recent analysis put forth the hypothesis that the lower glycolytic pathway shared by both ED and EMP pathways is able to sustain the highest flux when compared to other alternatives^22^. Absolute quantitative measurement of intracellular metabolite concentrations and fluxes by Park *et al*. revealed that the lower glycolysis has a higher overall driving force in terms of change in free energy (roughly six-fold higher) than currently stated in biochemistry textbooks^23^.

Concomitant to energy production and precursor synthesis hypotheses, recent studies have reaffirmed minimization of enzymes production as a key driver of optimizing resource allocation^24,25^. For example, fast-growing cells have to invest more resources for the synthesis of growth-related proteins (i.e., proteins associated with translational and transcriptional machinery)^25^. Instead of simply making proportionally more protein to accommodate higher growth requirements, they often shift metabolism towards pathways with more modest catalytic resources per unit of growth at the expense of energy efficiency^25,26^. Basan *et al*. further verified that *E*. *coli* switches from respiration to the more proteome-efficient fermentation under high growth rates^27^. Cellular metabolism has been shaped by evolution to ensure that carbon catabolic pathways are carefully selected to be in tune with both growth rate requirements and resource availability. Optimal glycolytic pathways must thus be able to balance high ATP production capacity while generating important intermediates and redox molecules at minimal proteome cost. These requirements are in direct conflict with one another requiring the establishment of Pareto optimal curves to decipher the relative “weights” between objectives that nature responds to when selecting different pathway designs.

**Figure 1:**
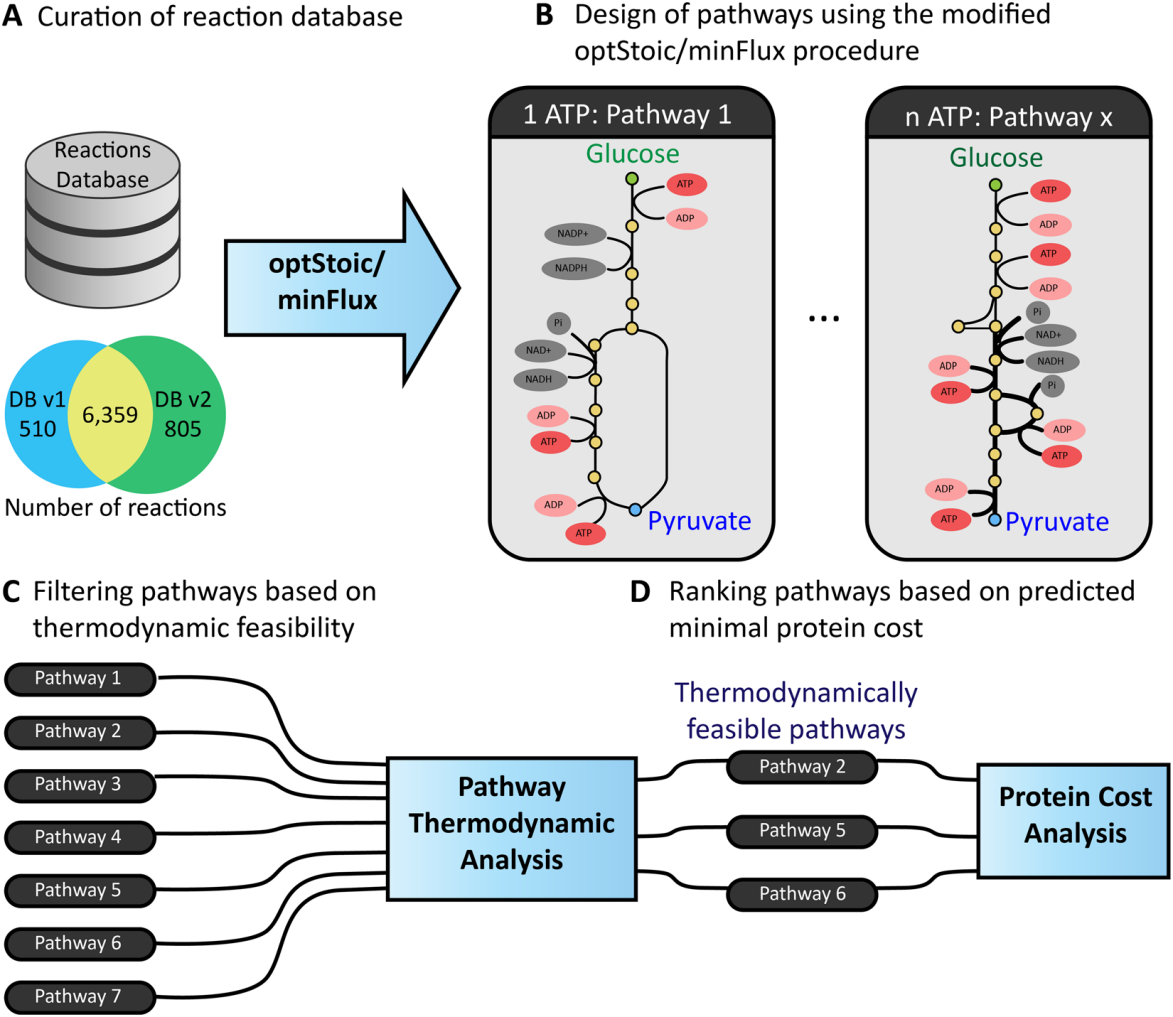
Schematic overview of the workflow for the design and analysis of glycolytic pathways. (**A**) The reaction database (DB v1) obtained from^29^ was curated and updated to DB v2 (see Supplementary File 1). (**B**) Pathways generating 1 to 5 ATP molecules were designed using the modified optStoic procedure. Visualization of the designed pathways was automated (see Methods). (**C**) The thermodynamic feasibility of each pathway under physiological metabolite concentration ranges was assessed using Max-min Driving Force (MDF) method. (**D**) The minimal protein cost for operating each thermodynamically feasible pathway was predicted using the Enzyme Cost Minimization (ECM) method^1^.

In this study, we aim to systematically assess the relative importance between various objectives driving pathway selection by exhaustively generating over 11,916 routes from glucose to pyruvate with varying energy production efficiency per mole of converted glucose and quantifying the corresponding total protein investment. The pathways were designed by combining annotated reactions from the entire chemical repertoire of organisms accessed from the KEGG^28^ database using a modified implementation of the optStoic protocol^29^ (Fig. 1). The Gibbs free energy of all reactions at standard conditions (i.e., 25 °C, pH 7, ionic strength of 0.1 M) was estimated using the Component Contribution method developed by Noor *et al*.^30^. Subsequently, the pathways were categorized based on their net ATP yield ranging from 1 to 5 mole of ATP per mole glucose and thereafter pruned to remove thermodynamically infeasible routes under physiologically relevant limits of intermediate metabolite concentrations. Minimal protein cost analysis on the feasible pathways revealed that the canonical ED and EMP pathways are indeed among the most protein cost-efficient pathways at physiological metabolite concentration ranges. Pathways with higher ATP yields were also identified (up to 5 ATP per glucose molecule). Driving thermodynamics closer to the limit (i.e., equilibrium) lowers the overall thermodynamic driving force thereby demanding a higher protein cost to drive the same amount of flux through the pathway. High (>2) ATP yielding pathways are also less tolerant to the changes in metabolite concentration ranges and ATP/ADP ratios, whereas the two canonical glycolytic pathways remain feasible under highly varying redox and metabolite states.

## Results

We first exhaustively traced pathways from glucose to pyruvate that conform to a general glycolysis stoichiometry while generating 1 to 5 ATP per glucose. We then filtered the pathways based on their thermodynamic feasibility and subsequently predicted the minimal protein cost of each pathway. We identified the Pareto optimal for the tradeoff between protein cost and ATP yield of glycolytic pathways and further determined the main factor(s) that affect the protein cost for a pathway. Several novel pathway designs with higher ATP yields are discussed and their lack of robustness to wide ATP/ADP fluctuations is demonstrated.

### Exhaustive enumeration of all glycolytic pathway variants using the modified optStoic procedure

A glycolytic pathway is defined here as the conversion of glucose into pyruvate accompanied by the generation of energy cofactor ATP and redox cofactor NAD(P)H. This conversion can be described by splitting the overall reaction into two balanced sub-reactions:

(i) Glucose + 2 NAD(P)^+^ = 2 Pyruvate + 2 NAD(P)H + 4 H^+^ (${{\rm{\Delta }}}_{r}{G^{\prime} }^{\circ }=-\,133.6\,$kJ/mol)
(ii) ADP + Phosphate + H^+^ = ATP + H_2_O (${{\rm{\Delta }}}_{r}{G^{\prime} }^{\circ }=26.4\,$kJ/mol)

The overall reaction allowing for a varying amount (denotes by coefficient *n*) of ATP produced is given by:

$$\begin{array}{c}{\rm{G}}{\rm{l}}{\rm{u}}{\rm{c}}{\rm{o}}{\rm{s}}{\rm{e}}+2\,{\rm{N}}{\rm{A}}{\rm{D}}{({\rm{P}})}^{+}+{\rm{n}}\,{\rm{A}}{\rm{D}}{\rm{P}}+{\rm{n}}\,{\rm{P}}{\rm{h}}{\rm{o}}{\rm{s}}{\rm{p}}{\rm{h}}{\rm{a}}{\rm{t}}{\rm{e}}\\ \,\,\,\,\,\,=2\,{\rm{P}}{\rm{y}}{\rm{r}}{\rm{u}}{\rm{v}}{\rm{a}}{\rm{t}}{\rm{e}}+2\,{\rm{N}}{\rm{A}}{\rm{D}}({\rm{P}}){\rm{H}}+{\rm{n}}\,{\rm{A}}{\rm{T}}{\rm{P}}+{\rm{n}}\,{{\rm{H}}}_{2}{\rm{O}}+(4-{\rm{n}}){{\rm{H}}}^{+}\end{array}$$

The maximum number of ATP that can be generated while maintaining ${{\rm{\Delta }}}_{r}{G^{\prime} }^{\circ }\le 0$ is therefore 133.6/26.4 = 5.06 mol/mol glucose. Although it is possible to generate additional ATPs through oxidative phosphorylation (e.g., up to 2.5 ATP/NADH)^31^, only ATP production through substrate-level phosphorylation from the glycolytic pathway is considered. We employed the optStoic procedure to prospect for pathways from a database of curated reactions derived from KEGG^28^ that perform the requisite conversion while generating from *n* = 1 to 5 mol ATP/mol glucose. The minFlux algorithm identifies minimal flux carrying network that conform to the given stoichiometry (see Methods). Alternate pathways were identified by iteratively appending integer cuts (see SI for details).

An important consideration for designing a glycolytic pathway is that ADP phosphorylation should be strictly coupled to it^19^. Using directly optStoic^29^ led to a significant number of pathways containing disjoint subnetworks such as ATP generating cycles (Supplementary Fig. S1A). Such disjoint subnetworks do not exchange carbon flux with the main glycolytic pathway chain. In extreme pathway analysis, such a closed loop of reactions that exchange only cofactors with other pathways is defined as Type II extreme pathway, whereas a thermodynamically infeasible closed loop that does not exchange any cofactors with its surroundings is defined as Type III^32^. We resolved this issue by appending the revised loopless-FBA constraints^33^ to the optStoic formulation (Supplementary Fig. S1B). In brief, the exchange reactions were first removed from the stoichiometric (*S*) matrix of the reaction database resulting in the *S*_*int*_ matrix which contains only the internal reactions. The rows involving cofactors (listed in Supplementary Table S1) were then removed to generate the *S*_*red*_ matrix. This step differs from the original loopless-FBA procedure that only eliminate Type III pathways, as we want to eliminate Type II internal cycles as well. The null basis (*N*_*red*_) of the *S*_*red*_ matrix is generated with each row indicating a closed loop of reactions that result in no net non-cofactor metabolite production. A disjoint subnetwork (i.e., Type II pathway) that exchanges only cofactors with the main glycolytic chain is now a closed loop on its own. The constraints (Methods, equations 7–11) derived from the loop law^33^ prevent net flux traversing any such a loop in a cyclic manner and essentially prohibit the selection of the disjoint subnetwork. Therefore, ATP production can occur only on the main carbon transfer pathway (i.e., glycolytic pathway) (Supplementary Fig. S1C).

As a result, a total of 11,916 unique glycolytic routes generating between one to five ATP without the undesirable disjoint subnetworks were identified (Supplementary Fig. S2, Supplementary Table S2). Both ED and EMP glycolytic pathways were also among them. The Jaccard similarity coefficient was used to verify that all the pathways generating the same ATP yield are indeed distinct from one another. The statistics of all pathways with respect to ATP yields, total flux (i.e., the minimum sum of the absolute values of fluxes) and total number of reactions are shown in Supplementary Fig. S2A,B.

### Imposing the thermodynamic feasibility test MDF and the effect of metabolite concentration ranges

**Figure 2:**
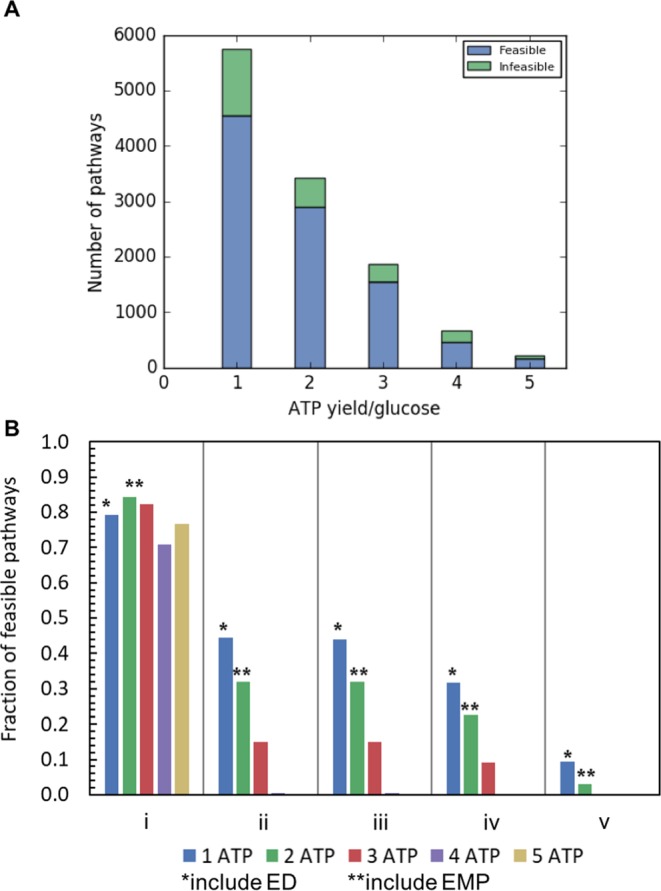
The number of pathways deemed thermodynamically feasible (**A**) after imposing the thermodynamic feasibility test MDF within the physiologically relevant concentration ranges (1 µM to 100 mM); (**B**) after imposing metabolite concentration conditions (i) to (v), see details in Supplementary Table S2A,B.

A glycolytic pathway variant may operate in the forward direction if and only if there is a positive thermodynamic driving force through each one of the constituting reactions (${\rm{i}}.\,{\rm{e}}.\,,{{\rm{\Delta }}}_{r}{G^{\prime} }_{j}\le 0,\forall j\in {{\boldsymbol{J}}}_{{\boldsymbol{path}}}$, where ${{\boldsymbol{J}}}_{{\boldsymbol{path}}}$ is a set of reactions in a pathway). Although all the glycolytic pathways designed above perform the overall conversion with a negative standard ${{\rm{\Delta }}}_{r}{G^{\prime} }^{\circ }$ (i.e., ${\sum }_{j\in {{\boldsymbol{J}}}_{{\boldsymbol{path}}}}{{\rm{\Delta }}}_{r}{G^{\prime} }_{j}^{\circ }\le 0$) and the directionality of each reaction is quantitatively assessed individually, it is not sufficient to ensure that all reaction steps *j* within the pathway can simultaneously have negative ${{\rm{\Delta }}}_{r}{G^{\prime} }_{j}$ for some intracellular metabolite concentrations. Consequently, we employed the max-min driving force (MDF) procedure^34^ to find if there is a set of metabolite concentrations within physiologically relevant ranges (1 µM to 100 mM, Supplementary Fig. S3) where all ${{\rm{\Delta }}}_{r}{G^{\prime} }_{j}$ are simultaneously negative. If at least one of the reaction steps has an unavoidable positive ${{\rm{\Delta }}}_{r}{G^{\prime} }_{j}$, then the pathway is deemed thermodynamically infeasible and is excluded from consideration. As a result, we were able to narrow down the solution pool by 19.3% (Fig. 2A and B, condition (i)). The imposition of the overall standard free energy of change negativity during pathway design seems to safeguard against thermodynamically infeasible designs with only a small fraction failing the more rigorous MDF test. Even though one would have expected that pathways that produce more ATP and thus are closer to the thermodynamic limit to involve a larger fraction of thermodynamically infeasible designs, we observed no such trend (Fig. 2A).

As expected the fraction of pathways deemed thermodynamically feasible strongly depends on the imposed metabolite concentration bounds. Tighter concentration bounds reduced the number of feasible pathways (Fig. 2B, Table S2A, Table S2B, condition (ii) to (v)). Pathways that produce more ATP are much more susceptible to the effect of concentration bound reduction. For example, when upper and lower limits obtained from experimental measurement of intracellular metabolite concentrations^23,35^ were imposed on ATP and ADP, none of the 5 ATP pathways were feasible (condition (ii)). When the concentration range of CO_2_ was also restricted based on experimental measurements^23,35^, twenty-four more 1 ATP-yielding pathways and one more 2 ATP-yielding pathways became infeasible (condition (iii)). In addition, when the cofactor ratios (i.e., ATP/ADP, NADPH/NADP, and NADH/NAD) were allowed to vary only within experimentally observed ranges (condition (iv)), 77% of the pathways designed were rejected including all of the 5 ATP yielding pathways. Finally, when the metabolite bounds were further restricted to between 1 µM to 10 mM (condition (v)), all of the 3 to 5 ATP-yielding pathways were found to be thermodynamically infeasible, whereas both canonical glycolytic pathways (i.e., EMP and ED) remained thermodynamically feasible. This strongly suggests that EMP and ED not only lie on the optimal tradeoff curve for yield vs. protein cost but do so while maintaining robustness to cofactor and metabolite concentration changes.

### The Pareto frontier of the tradeoff between protein cost and ATP yield

A significant fraction (from 10% to 20%) of the total proteome is allocated to glycolytic pathways (e.g., 10% to 15% in *E*. *coli*^25,26^ and 14% to 20% in yeast)^36,37^ to ensure the production of many intermediate metabolites and redox equivalents. We computationally explored whether it is possible to identify a glycolytic pathway variant with a lower cost than the canonical glycolysis (ED and EMP). The absence of a lower cost pathway would bolster the cost-benefit hypothesis that natural evolution converges toward parsimonious enzyme expression^38^. Often the sum of absolute fluxes through a pathway is used as a proxy to total enzyme requirement^39^, however, actual enzyme demand depends both on the enzyme catalytic efficiency and the metabolite concentrations. The lack of experimentally measured kinetic parameters, intracellular metabolite concentrations, and enzyme mechanisms hampers the development of a detailed mechanistic model for each pathway across different organisms.

To this end, we used the scalable convex optimization-based enzyme cost minimization (ECM) algorithm^1,40^ as a proxy for quantifying the effect of metabolite concentrations, kinetic parameters and Gibbs free energy on the enzyme demand per unit flux of a pathway. By recasting the reversible Michaelis-Menten equation as a separable rate law and integrating the Haldane relationship^40,41^, the kinetics associated with the reverse direction can be approximated by using Gibbs free energy of reaction (which can be predicted using Group Contribution method^42^ or Component Contribution method)^30^. We employed this computationally tractable approach to evaluate the minimal protein cost for operating any glycolytic pathway variants in a host cell-agnostic manner by assuming that all enzymes are equally efficient (see Methods) and metabolite concentrations are allowed to vary between 1 µM and 100 mM. Note that this analysis provides a lower bound to the actual enzyme cost^40^. Due to a large number of glycolytic variants, an automated pipeline was developed for the generation of the kinetic models of each thermodynamically feasible pathway (under the same condition) and the subsequent analysis of the minimal enzyme cost.

**Figure 3:**
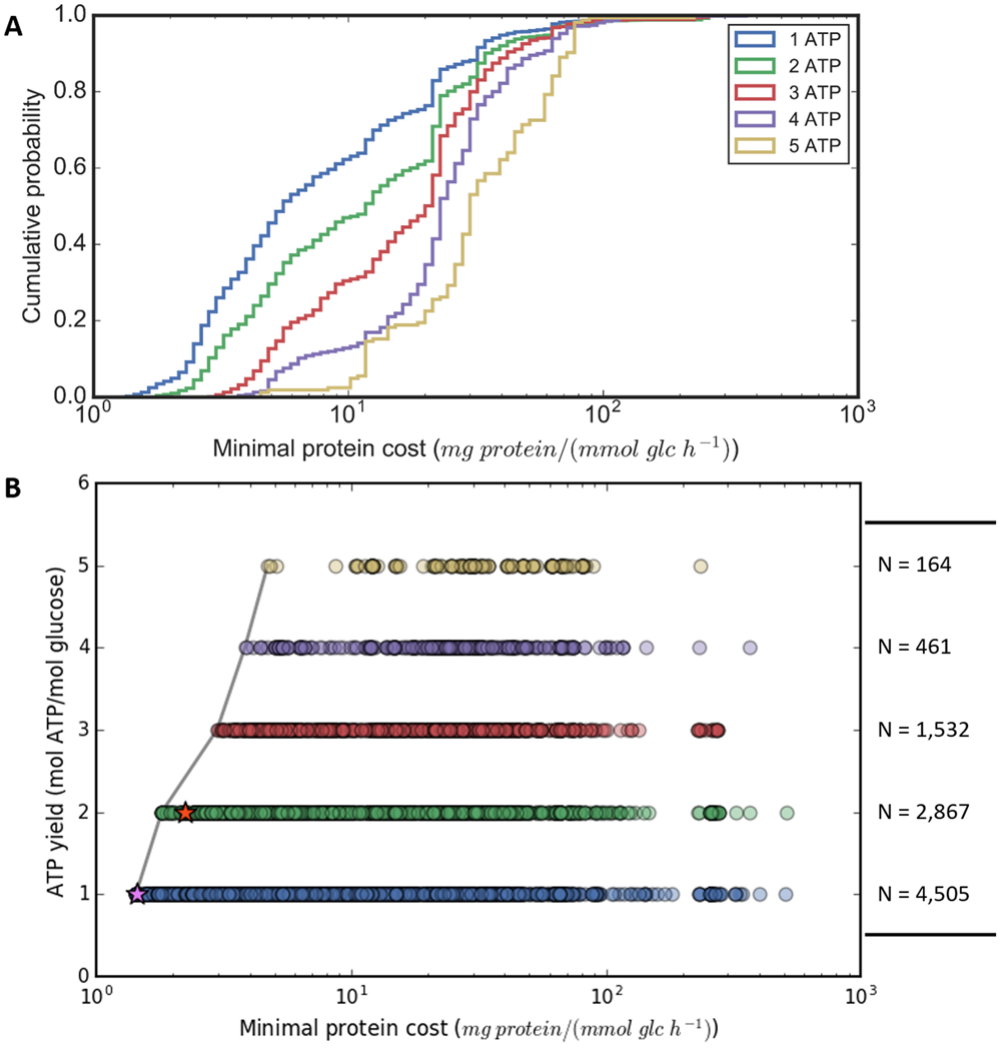
Pareto analysis of glycolytic pathway variants designed by the modified optStoic procedure. (**A**) The cumulative distribution function (CDF) plot of the minimal protein cost required to operate 1 to 5 ATP yielding glycolytic pathway variants. (**B**) The tradeoff plot between pathway ATP yield (mol ATP/mol glucose) and the minimal protein cost per unit glucose consumed. The grey line indicates the Pareto optimal of the tradeoff between ATP yields and protein cost of the glycolytic pathway. Pink and red stars indicate the ED and the EMP pathways, respectively. The number of data points (i.e., pathways) for each ATP yield category is described on the right of the plot. The lines and circles are color-coded based on the pathway ATP yield (see the legend in (**A**)).

The minimal protein cost of glycolytic pathway variants for each ATP yield spans a wide range regardless of the redox cofactor(s) generated (i.e., NADH or NADPH) (see Fig. 3A and Supplementary Fig. S4). By plotting the ATP yield (i.e., energetic objective) versus the minimal protein cost (i.e., operation cost) of all the glycolytic pathway variants (Fig. 3B), we derive the expected tradeoff between the two competing objectives. The Pareto frontier is constructed by connecting all the pathways with the least cost (i.e., Pareto optimal points) for each ATP yield value (Fig. 3B). The ATP yield of a glycolytic pathway on the Pareto optimum can only be increased at the expense of a higher investment in protein cost.

Notably, the canonical ED and EMP pathways lie close to the Pareto front suggesting that they are among the most protein cost-efficient pathways in their respective ATP yield category. The distance between the canonical ED pathway and the Pareto front is 0.129 mg Protein/mmol Glc/h, whereas the distance between the EMP pathway and the Pareto front is slightly larger at 0.429 mg Protein/mmol Glc/h. Out of the many possible ways (≥11,916) that nature can construct a glycolytic pathway, EMP and ED are consistent with a near-optimal protein resource allocation.

**Figure 4:**
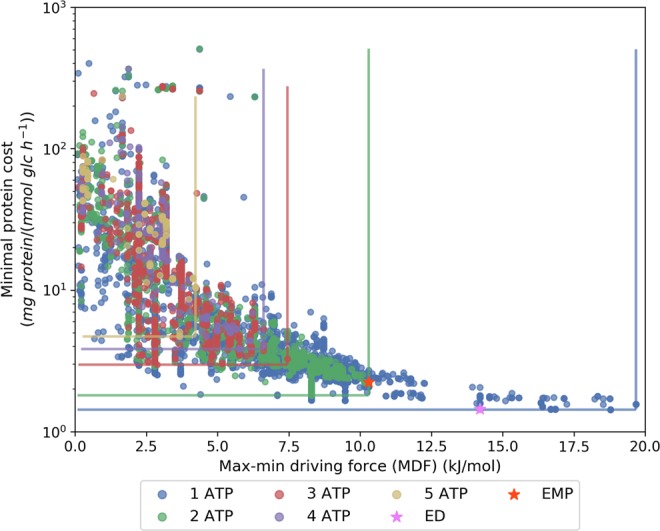
Identifying the key factors contributing to the protein cost of different ATP yielding pathways. The minimal protein cost correlates with the ${{\rm{\Delta }}}_{{\boldsymbol{r}}}{{\boldsymbol{G}}{\boldsymbol{^{\prime} }}}_{j}$ of the thermodynamic bottleneck (i.e., MDF) of a pathway under the assumption that all enzymes are equally fast and 1 mmol/gDW/h of glucose is converted to pyruvate. The vertical and horizontal lines show the ranges of minimal protein cost and MDF, respectively, of all pathways in each ATP yield category color-coded based on the ATP yield per unit glucose.

The minimal protein cost for a pathway is calculated using fairly wide concentration bounds (see Supplementary Table S2A,B, condition (i)). The analysis also assumes that all enzymes are equally fast with the same Michaelis constant (K_M_) for their respective substrates. Therefore, it is expected that the thermodynamic driving force for the thermodynamic bottleneck (i.e., indicated by the MDF objective) of a pathway is the main factor that affects protein cost (Fig. 4). Regardless of the required ATP yield, the minimal protein cost increases exponentially and asymptotically approaches infinity when the MDF objective of a pathway approaches zero (Fig. 4). This means that by having even just one reaction in a pathway with very high backward flux causes the protein cost of the entire pathway to dramatically increase. If the pathway has a large positive MDF function value, then the minimal protein cost required for the pathway is correspondingly small (Fig. 4). Overall, pathways generating higher ATP yield have a narrower range of MDF objective values than pathways with lower ATP yield, indicating the loss of driving force as the pathway has to conserve a higher fraction of energy from glucose to produce more ATP. The canonical EMP pathway has the second lowest MDF value among the 2 ATP-yielding pathways. The ED pathway is only 5.47 kJ/mol away from the 1 ATP yielding pathway with the minimal MDF.

### Pathways with a lower cost than the canonical glycolytic pathways

**Figure 5:**
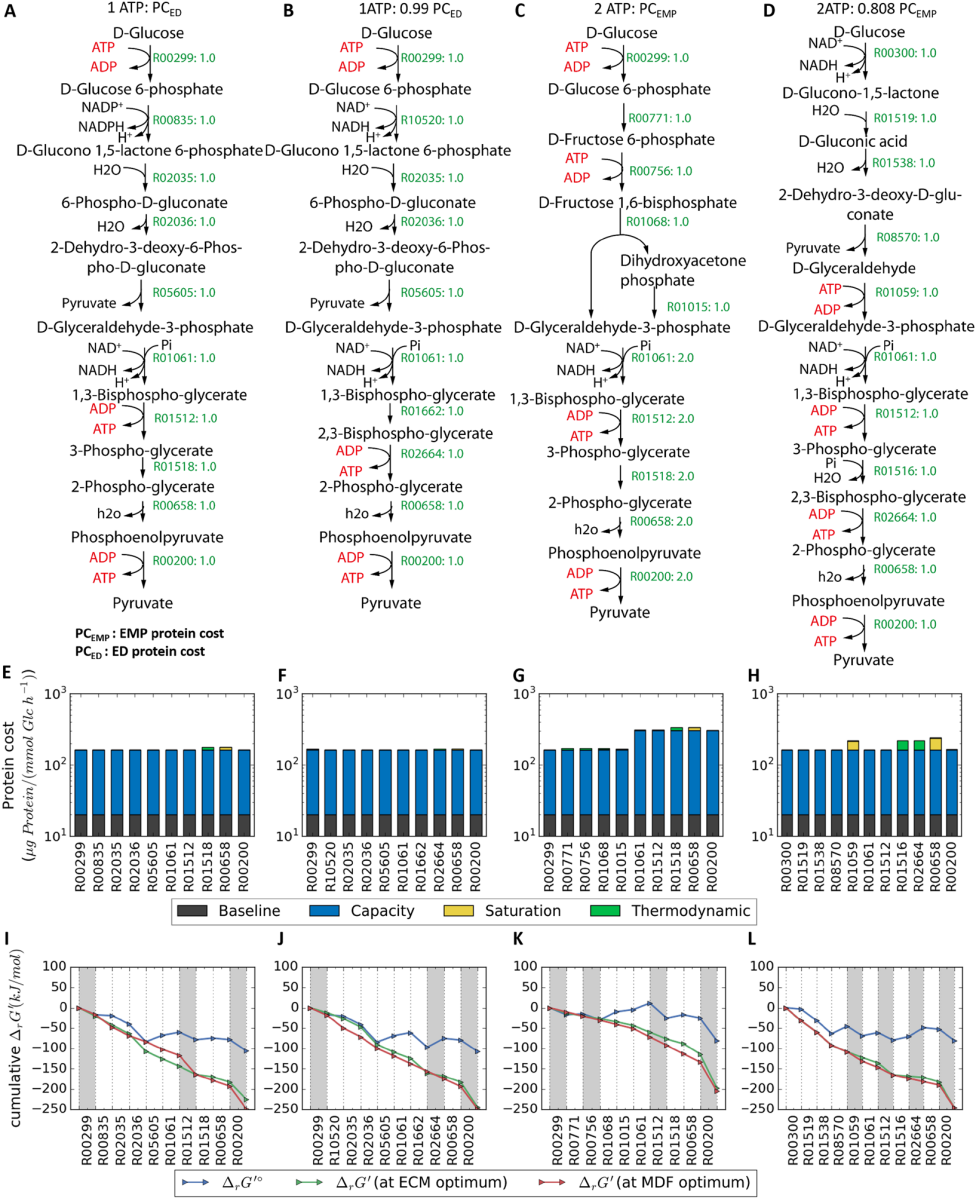
Pathway diagram designed using the modified optStoic procedure: (**A**) ED pathway, (**B**) a lower cost 1 ATP-generating pathway, (**C**) EMP pathway and (**D**) a lower cost 2 ATP-yielding pathway. The label beside each arrow represents the KEGG reaction ID and flux through each reaction. ATP and ADP cofactors are highlighted in red. (**E**–**H**) The distribution of protein cost through each pathway is displayed below each metabolic map. On each bar, the contribution of flux capacity, thermodynamic (i.e., high protein cost when the reaction is close to equilibrium causing backward fluxes) and enzyme saturation level (i.e., high protein cost when substrate concentration <K_M_) to the protein cost is represented by blue, green and yellow stacked bars, respectively. Note that the y-axis is in log_10_-scale as the contribution of each term is multiplicative. For this calculation, we assumed that all enzymes are equally fast (k_cat_) and have the same kinetic properties (K_M_), and the arbitrary baseline enzyme is set to 20 µg Protein/(mmol Glc /h). (**I**–**L**) The thermodynamic profile of each pathway expressed as transformed standard Gibbs free energy (blue line), transformed Gibbs free energy of reaction which accounts for the effect of metabolite concentrations when thermodynamic bottleneck (i.e., MDF) is minimized (red line) and when protein cost is minimized (green line). Regions shaded in grey highlight the reaction steps involving ATP.

We identified pathways that can operate at a lower cost for a given ATP yield value than the canonical pathways. The canonical ED pathway is ranked among the top ten pathways with the least protein cost. The least protein cost one-ATP generating pathway is very similar to the ED pathway (see Fig. 5A,B) affording only a 1% reduction in protein cost. It differs from the ED pathway in three reaction steps: (i) the glucose-6-phosphate dehydrogenase is NAD-dependent, (ii & iii) 1,3-bisphosphoglycerate (1,3-BPG) is converted into 2-phosphoglycerate (2-PG) through 2,3-bisphosphoglycerate (2,3-BPG) instead of 3-phosphoglycerate (3-PG) using a modified form of the Rapoport-Luebering (RL) bypass involving two enzymatic steps^43^. Other 2-PG kinases that could potentially interconvert between 2,3-BPG and 2-PG include that of *Methanothermus fervidus*^44^ and *Deinococcus radiodurans* have been demonstrated to catalyze only the phosphorylation of 2-PG to form 2,3-BPG but not the reverse^45^. However, since the ${{\rm{\Delta }}}_{r}G^{\prime}$ of reaction R02664 (2-PG + ATP = 2,3-BPG + ADP) can vary between −79 kJ/mol and 35 kJ/mol depending on the concentration of the participating metabolites, we considered it reversible. The 1% difference in protein cost is because phosphoglycerate mutase in the ED pathway has a relatively larger backward flux due to a less negative ${{\rm{\Delta }}}_{r}G^{\prime}$ rendering the enolase enzyme slightly less saturated by its substrate 2-PG. The top design solved this problem by using an alternative reaction (R02664: 2-PG + ATP = 2,3-BPG + ADP) with a more negative ${{\rm{\Delta }}}_{r}G^{\prime}$ (Fig. 5E,F,I,J).

The EMP glycolysis generates two ATP per glucose. It incurs 55% more protein cost than the ED pathway under condition i (Fig. 5C). The most protein cost-efficient 2 ATP generating-pathway, which requires about 80% of the protein cost required by EMP, relies on an ED-like pathway structure (Fig. 5D) because lower ED glycolysis requires less protein investment than lower EMP glycolysis (Fig. 5G,H,K,L). In this design, ATP usage and generation occur only in the lower glycolysis, while the upper glycolysis does not involve any ATP production or consumption. Such upper glycolysis is often derived from thermoacidophilic archaebacteria (e.g., *Sulfolobus solfataricus* and *Thermoplasma acidophilum*)^46^. ATP is first invested in the phosphorylation of glyceraldehyde that is generated from the cleavage of KDG by KDG aldolase. Three ATPs are then produced downstream through the conversions of 1,3-BPG to 3-PG, 2,3-BPG to 2-PG and PEP to pyruvate. This is made possible by the investment of inorganic phosphate to phosphorylate 3-PG to form the higher energy intermediate 2,3-BPG. The lack of phosphorylation of glucose to minimize the escape (i.e., diffusion) of neutral charged glucose from the cell in the first step possibly made this pathway less favorable for most organisms. Nevertheless, the second step converts glucono-1,5-lactone into gluconic acid which is a polar compound with reduced membrane permeability^19^.

### Pathways generating higher ATP yield than the canonical glycolytic pathways

**Figure 6:**
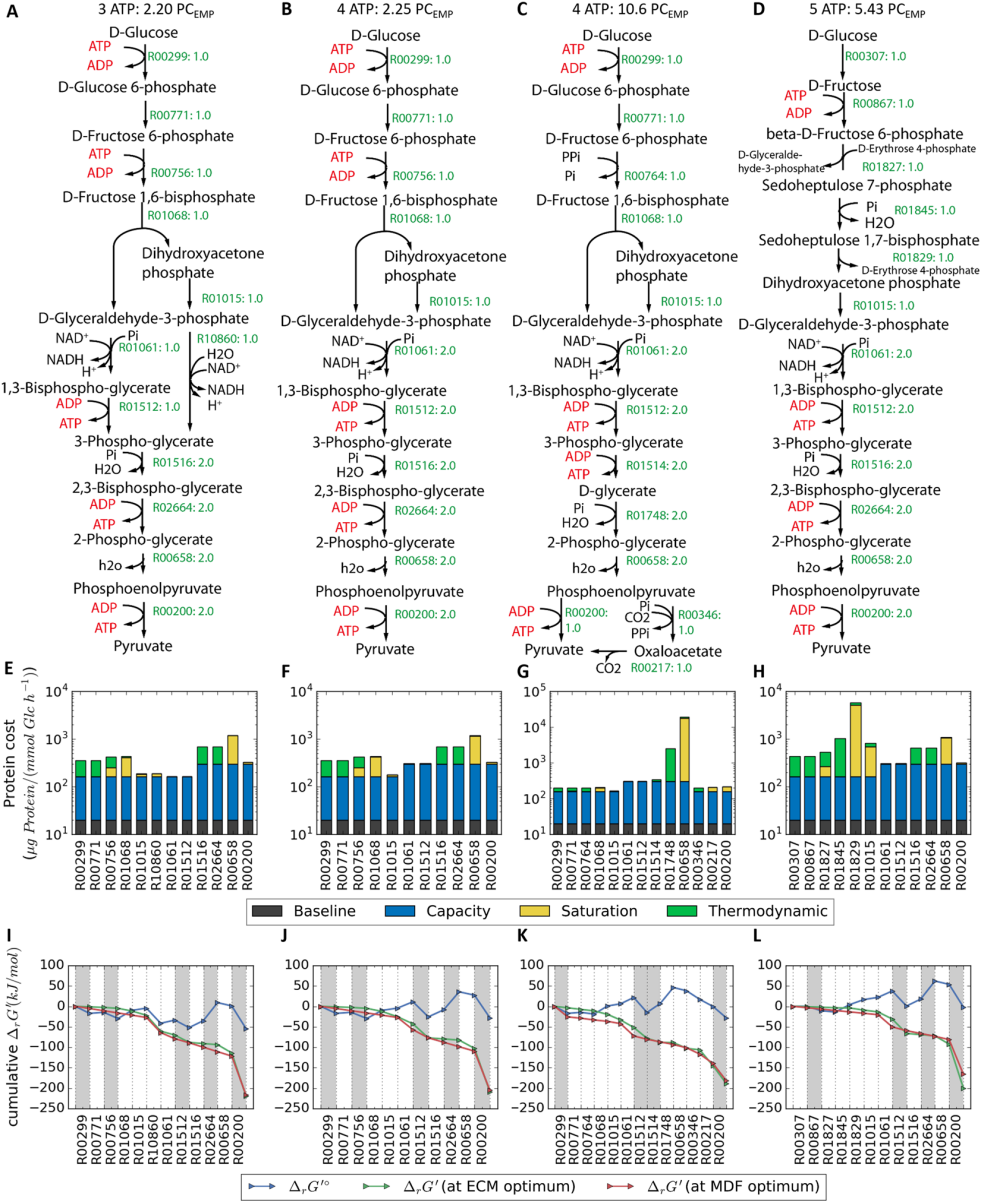
Pathway generating 3 to 5 ATP designed using the modified optStoic procedure: (**A**) 3ATP pathway, (**B**) 4 ATP pathway A, (**C**) 4 ATP pathway B and (**D**) 5 ATP pathway. (**E**–**H**) The distribution of protein cost through each pathway is displayed below each metabolic map. (**I**–**L**) The thermodynamic profile of each pathway. All the figure labels and legends are the same as mentioned in Fig. 5.

In this section, we will discuss a few identified pathways generating more than two ATPs per glucose molecule. To derive an extra ATP from a pathway resembling EMP, the carbon flux from GAP is first split into two branches (Fig. 6A). While one of the GAP molecules traverses through the typical NAD^+^-dependent phosphorylating GAP dehydrogenase and ATP-generating phosphoglycerate kinase, the other bypasses the ATP-forming phosphoglycerate kinase step by using the non-phosphorylating GAP dehydrogenase (GAPN). Both NAD^+^ and NADP^+^-dependent GAPN have been previously identified in hyperthermophilic archaea *T*. *tenax* as well as in photosynthetic higher eukaryotes^47^. The resulting 3-PG is routed through the modified Rapoport-Luebering shunt (described in the previous section) to form 2-PG, thereby generating two extra ATPs. Five ATPs are produced in lower glycolysis which compensates for the two ATPs invested in the upper glycolysis for a net of three ATPs.

A net of 4 ATPs can be generated from a pathway shown in Fig. 6B. In this pathway, the upper glycolysis is similar to the EMP pathway wherein 2 ATPs are consumed and GAP is converted to 1,3-BPG. Subsequently, the lower glycolysis generates 6 ATPs through a subnetwork similar to that of 2 ATP-yielding pathway in Fig. 5D but with double the flux. Another interesting 4 ATP-yielding pathway design mimics the *C*. *cellulolyticum*^48^ glycolysis by utilizing a PP_i_-dependent PFK thereby bypassing an ATP investment upstream requirement (Fig. 6C). 5 ATPs are generated in the lower glycolysis yielding a net of 4 ATPs. *C*. *cellulolyticum*, an obligate anaerobe, could generate up to 5 NTP molecules (i.e., ATP and GTP) per glucose molecule through its glycolytic pathway. This is achieved by (i) using a reversible PP_i_-dependent PFK; (ii) generating 2 GTPs from the reactions catalyzed by phosphoglycerate kinase (1,3-BPG + GDP = 3-PG + GTP) and a GTP-dependent PEP carboxykinase (EC 4.1.1.32: PEP + GDP + CO_2_ = oxaloacetate + ATP); and (iii) generating 2 ATPs from an ATP-dependent pyruvate carboxylase (oxaloacetate + ADP = pyruvate + ATP + CO_2_). The PP_i_ consumed by PFK is suggested by Rabinowitz *et al*. to be regenerated through the conversion of sedoheptulose-1,7-bisphosphate (SBP) to sedoheptulose 7-phosphate (S7P)^47^. Accounting for PP_i_ usage, this *C*. *cellulolyticum* pathway generates an equivalent of 4 ATPs. In the pathway designed using the modified optStoic procedure (Fig. 6C), the PP_i_ is recouped at the end of the pathway through the conversion of PEP to oxaloacetate. Note that the actual *Clostridial* glycolysis relies on GTP, which is absent in this design. However, the pathway shown in Fig. 6C requires almost ten times more protein than the EMP pathway to operate at the same glucose conversion flux due to the large backward flux of the reaction involving phosphorylation of glycerate by organic phosphate towards 2-phosphoglycerate. A ^13^C-MFA study on *C*. *cellulolyticum* showed that significant backward flux is observed in the upper glycolysis due to its utilization of PP_i_-dependent PFK instead of the irreversible ATP-dependent PFK, which possibly leads to a lower net forward flux through the pathway^48^.

Finally, a 5 ATP yielding pathway bypasses an ATP investment in the upper glycolysis by combining transaldolase from the non-oxidative pentose phosphate pathway with the reverse of the Calvin cycle (Fig. 6D). It first converts fructose-6-phosphate and erythrose-4-phosphate (E4P) into GAP and S7P. The latter is then phosphorylated by the reversible sedoheptulose bisphosphatase^49^ to form SBP. SBP is subsequently converted into dihydroxyacetone phosphate (DHAP) and E4P, which is catalyzed by fructose-bisphosphate (FBP) aldolase. The latter returns to the previous steps, while DHAP is channeled into the lower glycolysis. The lower glycolysis is similar to the 4 ATP pathway in Fig. 6B, which generates 6 ATPs. Hence, the net ATP yield is 5 ATPs after subtracting the ATP invested for fructose phosphorylation. The sedoheptulose bisphosphatase operates with a significant backward flux, which leads to the significantly high non-saturation of the FBP aldolase. In this way, the pathway is able to retain more free energy for ATP production.

Comparison of the protein cost distribution between the low ATP (Fig. 5E–H) and high ATP-yielding pathways (Fig. 6E–H) reveals that pathways with higher ATP yields are generally comprised of reactions with much higher backward flux as well as lower saturation levels (i.e., substrate concentration <<K_M_). A reaction close to equilibrium is often followed by a reaction that is substrate significantly sub-saturated. Even though the overall pathway ${{\rm{\Delta }}}_{r}{G^{\prime} }^{\circ }$ (standard free energy change) is closer to zero for higher ATP-yielding pathways (Fig. 6I–L), the overall ${{\rm{\Delta }}}_{r}G^{\prime}$ can be lowered towards negative values by optimizing the metabolite concentrations to minimize the thermodynamic bottleneck of a pathway. Generally, for higher ATP yields, the energy content in glucose is routed over many more reaction steps (in particular ATP generating steps), thereby causing more reactions to undergo a smaller drop in ${{\rm{\Delta }}}_{r}G^{\prime}$(Fig. 6I–L). Near-equilibrium values of ${{\rm{\Delta }}}_{r}G^{\prime}$ imply a lower flux through the pathway (i.e., low ATP production flux), which could be detrimental to growth if glycolysis is the major ATP generating mechanism. In addition, higher ATP yielding pathways are much more likely to become thermodynamically infeasible when the bounds on metabolite concentrations are tightened (Supplementary Table S2). This implies that higher ATP yielding pathways can access a much smaller range of allowable metabolite concentrations. Therefore, a high ATP yielding pathway that may be feasible in a slow growing organism *C*. *cellulolyticum*, may become infeasible in a different organism with a higher growth rate and/or different intracellular metabolite pool. Overall, a combination of higher protein cost, lower pathway driving force and sensitivity to metabolite pools may explain why these higher ATP yielding pathways have not become dominant in glycolytic organisms. In addition, instead of optimizing ATP yield, organisms may choose to maximize the rate of ATP generation by increasing substrate uptake rate through lower yield pathways^50^.

### The canonical glycolytic pathways are robust to changes in ATP/ADP ratio

**Figure 7:**
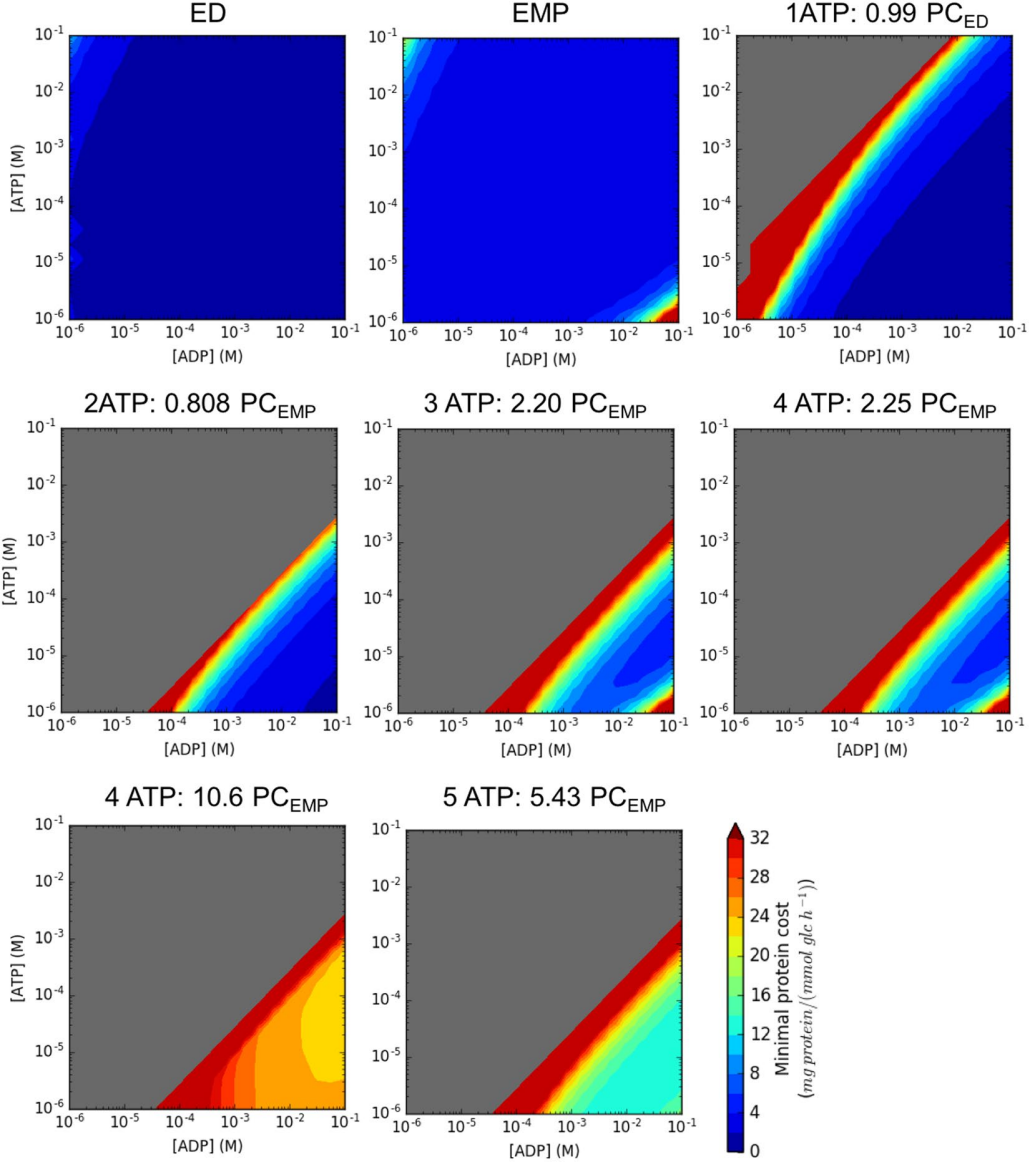
The effect of ATP and ADP concentrations on the pathway thermodynamic feasibility and minimal enzyme cost of glycolytic pathway variants shown in (**A**–**F**). 400 pairs of ATP and ADP concentrations were sampled uniformly from the log concentration ranges. The MDF analysis and ECM analysis were performed on each pathway when ATP and ADP concentrations were constrained to the sampled values. Grey color regions indicate that the pathway is thermodynamically infeasible. The color scales according to the minimal protein cost. Values above 32 mg protein/mmol glc/h are set to dark red color.

We explored the feasibility of the identified glycolytic pathways for different ATP/ADP ratio ranges based on MDF and ECM analyses. We uniformly sampled 400 pairs of values of ATP and ADP. For each pair of ATP/ADP concentrations, we assessed pathway thermodynamic feasibility and minimal protein cost. Figure 7 shows that the canonical glycolytic pathways ED and EMP remain thermodynamically feasible across all ATP/ADP concentration values, unlike other higher ATP yielding or lower protein cost pathways. In particular, the ED pathway maintains a low protein cost across the entire range of ATP/ADP concentrations. This is surprising as one may expect that a glycolytic pathway would not be feasible when the ATP concentration is much higher than that of ADP, which would lower the driving force for ATP synthesis. Based on Fig. 7, higher ATP yielding pathways remain feasible only when the ATP/ADP ratio is less than one. However, ATP/ADP ratios measured in mammalian cells, yeast or *E*. *coli* are all above unity^23,51,52^. We repeated the MDF test when the ATP/ADP ratio is constrained to be above unity (Supplementary Table S2, condition (vi)). We found that all 5 ATP yielding pathways become infeasible and only four 4 ATP yielding pathways and just 15% of the 3 ATP yielding pathways remained feasible. The robustness of ED and EMP pathways for such a wide range of ATP/ADP concentrations suggest that they could operate in the direction of glycolysis even under a significant perturbation of the ATP/ADP pool (e.g., due to stress or variable substrate supply).

Nevertheless, we did identify one 1-ATP yielding and four 2-ATP yielding glycolytic pathways with lower protein cost than ED and EMP that remained feasible across a broad range of the ATP/ADP ratio (Supplementary Figs S5 and S6). Notably, these pathways were nearly identical to the ED or EMP pathway except for the selection of alternate redox cofactors. For example, the modified ED pathway (Supplementary Fig. S7A) utilizes an NAD-dependent glucose-6-phosphate dehydrogenase whereas the two modified EMP pathways (Supplementary Fig. S7D and Supplementary Fig. S7E) produce NADPH instead of NADH when converting GAP to 1,3-BPG. In addition, the first three steps shown in Supplementary Fig. S7B–E bypass the hexokinase step and convert glucose to fructose using fructose-isomerase. Such pathways are likely unfavorable because the phosphorylation of glucose in the first glycolytic step is key to preventing it from escaping the cellular membrane. In addition, fructose-isomerase has broad substrate specificity, which makes it less desirable for glucose uptake when compared with the hexokinase in the EMP pathway.

## Discussion

Flamholz *et al*. previously hypothesized that there exists a tradeoff between ATP yield and protein cost by showing that the ED pathway requires a lower enzyme cost than the EMP pathway^1^. However, it was unclear as to where the two pathways stand in terms of their cost efficiency when compared to glycolytic alternatives that yield the same number of ATP molecules. We attempted to answer the question by exhaustively analyzing a large number of synthetic glycolytic variants generated computationally using the modified optStoic procedure and screened by the MDF^34^ and ECM methods of analysis^1,40^. Our simulation results suggested that the dominance of the canonical ED and EMP pathways is indeed consistent with the minimization of the overall protein cost hypothesis. Other natural semi-phosphorylative ED variants are also found to exhibit comparable properties to the canonical ED pathway (Fig. S8). However, many other pathway alternatives were identified sharing the same (or even better) protein cost economy. In addition, many more were identified that could generate more than 2 ATP molecules (up to 5) per glucose molecule. It appears that the key distinguishing feature of the canonical EMP and ED pathways is the fact that they could operate for a wide range of metabolite concentrations and ATP/ADP ratios unlike the majority of all other glycolytic alternatives (over 67% of them).

While optimizing protein resource is an important factor in pathway selection, robustness to extracellular changes (e.g, high/low glucose supply, alternate carbon substrates, stress, etc.) also plays a major role in shaping metabolic pathways^53^. Furthermore, pathways need to retain operability beyond the realms of exponential (i.e., steady-state) growth phase, where cofactors and intracellular metabolites in organisms undergo high fluctuations (e.g., during lag, stationary, cell-division phases, etc.)^54,55^. We find that the robustness of a pathway to different intracellular concentrations may ultimately determine whether a pathway is universally adopted. We have demonstrated that at least for glycolytic pathways, the Pareto optimal surface for protein cost vs. energetic yield must be extended to include the dimension of robustness. It is plausible that robustness imperatives that apply to naturally selected pathways may also be important for synthetic ones. This may explain why it is important to *a posteriori* subject engineered cells to directed evolution^56^, conditioning^57^ or adaptive evolution^58^ to identify variants with robust phenotypes. Interestingly, evolved strains often alleviate these bottlenecks with mutations that confer robustness to the engineered pathway (e.g., increase overall pathway carbon flow, increase enzyme catalytic efficiency to overcome unfavorable thermodynamics, and reduce production of unnecessary enzymes and competing by-products) and subsequently growth rate^58^. Alternatively, one may impose feasibility of operation of a pathway over a wide range of concentrations and relevant energetic and redox cofactor ratios (i.e., ATP/ADP, NAD(P)H/NAD(P)) as a design imperative. Our procedure can be used in this case to facilitate the design, analysis, and down-selection of (synthetic) bioconversion pathways.

In this study, we assume that the metabolite concentrations are homogeneous within a cell. Compartmentation of metabolites and proteins may affect the optimal metabolite concentration for MDF (i.e., pathway feasibility) as well as the protein cost required to operate the pathway. For example, cells may need specific proteins/energetic demand to transport the metabolites between compartments. However, it is possible that with compartmentations, multiple local concentrations of a metabolite are allowed in different compartments and a pathway that was infeasible may become feasible. In addition, the concentration of metabolites varies significantly across species. This would ultimately affect the feasibility of various glycolytic alternatives. Due to the difficulty of obtaining high quality absolute quantitative metabolomics data for a large number of species, we have decided to perform these *in silico* experiments using the widest possible range of metabolite concentrations.

Beyond the current focus of this study, this work provides templates for designing alternate glycolytic pathways for potential metabolic engineering applications (e.g., by re-routing glycolytic intermediates or making higher energy yield), especially for metabolites near central carbon metabolism that require additional ATP for biosynthesis. While most of the designed glycolytic pathways generating >2 ATP become thermodynamically infeasible, we did identify several alternatives that can be coupled with other engineered pathways for improving overall yield, albeit with higher protein cost and lower flux rates. This introduces a separate Pareto optimality problem between yield and productivity and designing pathways to systematically optimize between them. For example, we identified multiple such high ATP yielding glycolytic pathways (see Fig. 6) that resemble the one that obligate anaerobes such as *C*. *cellulolyticum* and *C*. *thermocellum* utilize^13,59^. Recent efforts have started to leverage these organisms for the biosynthesis of various biofuels and bioproducts^60,61^.

## Methods

### Update of the optStoic reaction database

The reaction database for the optStoic procedure^29^ was curated to ensure that all reactions are elementally (i.e., C, O, N, P, and S) balanced and updated with new reactions from the KEGG database^28^. The updated optStoic reaction database contains a total of 7,164 reactions and 5,969 metabolites (see Supplementary Data File 1).

Reactions that are incomplete (e.g., elementally imbalanced or contain generic stoichiometric coefficient (e.g., R05327: n C00043 + n C00167 = C00518 + 2n C00015)) were removed. The standard transformed Gibbs free energy of all reactions (${{\rm{\Delta }}}_{r}{G^{\prime} }_{j}^{^\circ }$) at pH 7, 25°C and ionic strength of 0.1 M was estimated using the Component Contribution method^30^. For metabolites that were not present in the Component Contribution Python package, the chemical structure (i.e., Molfile) was retrieved from KEGG and converted into InChI using Open Babel^62^ or ChemAxon (Marvin 16.7.18, 2016, ChemAxon (http://www.chemaxon.com)). The Gibbs free energy of formation of these compounds was then calculated using the same method and added to the database. Note that the same standard Gibbs free energy change of a reaction (${{\rm{\Delta }}}_{r}{G^{\prime} }_{j}^{^\circ }$) estimated here is also used for the formulations of max-min driving force and enzyme cost minimization. The reaction directionality was determined by assessing the impact of the sign on the free energy of change by either depleting the product or reactant^63^.

*Step 1:* ${{\rm{\Delta }}}_{r}{{G}^{{\rm{^{\prime} }}}}_{j,min}={{\rm{\Delta }}}_{r}{{G}^{{\rm{^{\prime} }}}}_{j}^{{}^{\circ }}+RT\,{\rm{l}}{\rm{n}}\,{Q}_{min}$ is calculated at substrate concentrations of 0.1 M and product concentrations of 1 µM,

*Step 2:* ${{\rm{\Delta }}}_{r}{{G}^{{\rm{^{\prime} }}}}_{j,max}={{\rm{\Delta }}}_{r}{{G}^{{\rm{^{\prime} }}}}_{j}^{{}^{\circ }}+RT\,{\rm{l}}{\rm{n}}\,{Q}_{max}$ is calculated at substrate concentrations of 1 µM and product concentrations of 0.1 M,

*Step 3:* A reaction is deemed (i) irreversible in the forward direction if both ${{\rm{\Delta }}}_{r}{{G}^{{\rm{^{\prime} }}}}_{j,min} < 0$ and ${{\rm{\Delta }}}_{r}{{G}^{{\rm{^{\prime} }}}}_{j,max} < 0$, (ii) irreversible in the reverse direction if both ${{\rm{\Delta }}}_{r}{G^{\prime} }_{j{,}\min } > 0$ and ${{\rm{\Delta }}}_{r}{G^{\prime} }_{j{,}\max } > 0$, and (iii) reversible if ${{\rm{\Delta }}}_{r}{{G}^{{\rm{^{\prime} }}}}_{j,min}\le 0$ and ${{\rm{\Delta }}}_{r}{{G}^{{\rm{^{\prime} }}}}_{j,max}\ge 0$. If ${{\rm{\Delta }}}_{r}{G^{\prime} }_{j}^{^\circ }$ cannot be approximated (e.g., due to the absence of standard Gibbs free energy of formation for at least one of the reactants), then the reaction is assumed to be reversible.

The directionality of 204 reactions, particularly those involving ATP, were manually curated based on Chowdhury and Maranas^29^. Consequently, the updated database contains a total of 5,014 reversible reactions (out of which 1,898 reactions have undefined ${{\rm{\Delta }}}_{r}{G^{\prime} }_{j}^{^\circ }$) and 2,150 irreversible reactions.

### Designing pathways using the modified optStoic procedure

The overall stoichiometry of glycolysis allowing for a varying amount of produced ATP moles (i.e., n) per glucose mole is given by:

1 $$\begin{array}{c}{\rm{G}}{\rm{l}}{\rm{u}}{\rm{c}}{\rm{o}}{\rm{s}}{\rm{e}}+2\,{\rm{N}}{\rm{A}}{\rm{D}}{({\rm{P}})}^{+}+{\rm{n}}\,{\rm{A}}{\rm{D}}{\rm{P}}+{\rm{n}}\,{\rm{P}}{\rm{h}}{\rm{o}}{\rm{s}}{\rm{p}}{\rm{h}}{\rm{a}}{\rm{t}}{\rm{e}}\,\,\,\\ =\hspace{.25pt}\,2\,{\rm{P}}{\rm{y}}{\rm{r}}{\rm{u}}{\rm{v}}{\rm{a}}{\rm{t}}{\rm{e}}+2\,{\rm{N}}{\rm{A}}{\rm{D}}({\rm{P}}){\rm{H}}+{\rm{n}}\,{\rm{A}}{\rm{T}}{\rm{P}}+{\rm{n}}\,{{\rm{H}}}_{2}{\rm{O}}+(4-{\rm{n}}){{\rm{H}}}^{+}\end{array}$$

This overall reaction is henceforth denoted as the design reaction. We use the minFlux mixed-integer linear programming (MILP) formulation from the optStoic procedure^29^ to identify the set of reactions that conform to the above glycolytic stoichiometry. The minFlux formulation is given by

2 $$minimize{\sum }_{j\in {\boldsymbol{J}}\backslash {{\boldsymbol{J}}}_{{\boldsymbol{exchange}}}}|{v}_{j}|\,\,\,(min\,Flux)$$

3 $$subject\,to\sum _{j\in {\boldsymbol{J}}}{S}_{ij}{v}_{j}=0,\forall \,i\in {\boldsymbol{I}}$$

4 $${v}_{i}^{EX}={q}_{i},\,\forall \,i\in {{\boldsymbol{I}}}_{{\boldsymbol{stoich}}}$$

5 $$L{B}_{j}\le {v}_{j}\le U{B}_{j},\,\forall \,j\in {\boldsymbol{J}}\,$$

$${v}_{j}\in {\mathbb{Z}}\,\vee \,{\mathbb{R}},{v}_{i}^{EX}\in {\mathbb{Z}}\,\vee \,{\mathbb{R}}$$

where set ***I*** and ***J*** represent metabolites and reactions, respectively. ${{\boldsymbol{I}}}_{{\boldsymbol{stoich}}}$ is a set of metabolites that participate in the design reaction. ${S}_{ij}$ is the stoichiometric matrix with each row representing a metabolite *i* and each column representing reaction *j*, *v*_*j*_ is the flux of reaction *j*, ${v}_{i}^{EX}$ is the exchange reaction for metabol*i*te *i*. The set of all exchange reactions ${v}_{i}^{EX}$ is declared as ${{\boldsymbol{J}}}_{{\boldsymbol{exchange}}}$. Both *v*_*j*_ and ${v}_{i}^{EX}$ can either be integers (${\mathbb{Z}}$) or real numbers (${\mathbb{R}}$). Parameter *q*_*i*_ is the stoichiometric coefficient of metabolite *i* in the design reaction. $L{B}_{j}$ and $U{B}_{j}$ indicate the lower and upper bounds on the flux of the respective reaction *j*. The objective function (equation 2) ensures that the sum of the absolute flux through the entire network of reactions is minimized. Constraint 3 ensures stoichiometric (mass) balance for all metabolite *i* in the network. Constraint 4 enforces that the flux through exchange reaction for all metabolites given in the design reaction is proportional to their stoichiometric coefficient. Constraint 5 imposes upper and lower bound on the *v*_*j*_ based on the reaction directionality as discussed in the previous section.

However, the original minFlux formulation does not restrict the identification of disjoint subnetworks that are only connected with the primary carbon transfer pathway (glycolysis in this case) with only energy (e.g., ATP), redox (e.g., NAD(P)H) or other cofactors (e.g., H_2_O) exchanges. This often results in pathway designs where the entire driving force of the conversion is accomplished by futile cycles disconnected from the main metabolism (Supplementary Fig. S1A). For example, a pathway shown in Supplementary Fig. S1A contains two undesirable subnetworks and one of them operates in the direction of ATP generation. This issue was remedied here by using an approach similar to the loopless-FBA^33^ to eliminate the subnetworks.

*Step 1*: An internal stoichiometric matrix (*S*_*int*_) was constructed by first removing all exchange reactions (columns). Subsequently, rows containing the selected cofactors (Supplementary Table S1) were removed resulting in the ${S}_{red}$ matrix.

*Step 2*: The rational basis of the null space of the *S*_*red*_ matrix is calculated resulting in the *N*_*red*_ matrix. Consequently, all loops of reactions whose net conversion results in only cofactors consumption or generation such as (i) ATP hydrolysis, (ii) redox generation or (iii) water-splitting, can be represented by linear combination of the null basis *N*_*red*_.

*Step 3:* The following constraints are then appended to the optStoic formulation:

6 $$\sum _{j}{N}_{red,jl}^{T}{G}_{j}=0,\,\forall \,l\in {\boldsymbol{L}}$$

7 $${G}_{j}\ge -\,M{a}_{j}+(1-{a}_{j}),\,\forall \,j\in {\boldsymbol{J}}$$

8 $${G}_{j}\ge -\,{a}_{j}+M(1-{a}_{j}),\,\forall \,j\in {\boldsymbol{J}}$$

9 $${v}_{j}\ge -\,M(1-{a}_{j}),\,\forall \,j\in {\boldsymbol{J}}$$

10 $${v}_{j}\le M{a}_{j},\,\forall \,j\in {\boldsymbol{J}}$$

$${G}_{j}\in {\mathbb{R}};{a}_{j}\in \{0,\,1\}$$

where ***L*** is the set of all loops, ${N}_{red}^{T}$ is the transpose of the null basis ${N}_{red}$with loop *l* and reaction *j*, *M* is a large positive number (*e*.g., 1000), and the variable *G*_*j*_ is a pseudo free energy parameter^33^, which does not reflect the actual thermodynamic driving force equivalent of reaction *j* (i.e., ${{\rm{\Delta }}}_{r}{G^{\prime} }_{j}$). Constraint 6 imposes the loop law^33^ wherein the sum of *G*_*j*_ for all reactions in a closed loop *l *has to be zero thereby preventing all the reactions in the loop to carry flux simultaneously in a cyclical manner. Constraints 7 and 8 ensure that *G*_*j*_ is strictly positive ($1\le {G}_{j}\le M$) or negative ($-\,M\le {G}_{j}\le -\,1$) so that the solution ${G}_{j}=0$ can be avoided. The binary variables *a*_*j*_ are introduced in constraints 7 to 10 to ensure that ${v}_{j} > 0$ when ${G}_{j} < 0$ and vice versa. By adding these constraints, a feasible solution that is a network devoid of the undesirable subnetwork can be identified (Supplementary Fig. S1C).

Integer cut constraints are then introduced to exhaustively identify alternate optimal pathways that satisfy the design equation. The MILP problems were solved using the CPLEX v.12.6.1 solver accessed through the GAMS (v24.4.1) modeling system and Gurobi Optimizer v6.5.1 using Python 2.7. The codes for the optStoic procedure are provided as a part of the *optstoic-python* Python package that was developed in this study and can be downloaded from https://github.com/maranasgroup/optstoic-python and www.maranasgroup.com. The pathways designed in this study are provided in JSON format (see Supplementary Data Files 2–6).

### Assessing the thermodynamic feasibility of a pathway

The thermodynamic feasibility of each pathway under physiological concentration ranges are assessed using the max-min driving force (MDF) formulation^34^. The MDF formulation in essence attempts to identify a set of metabolite concentrations that ensure the lowest free energy changes for all the reactions in a pathway. If the objective value of MDF is positive, then the pathway is thermodynamically infeasible. See details in SI supplementary text.

### Protein cost analysis

The minimal enzyme demand in units of mg protein/mmol glucose/h for each one of the thermodynamically feasible pathways is then estimated based on the enzyme cost minimization (ECM) method^1,40^. See details in SI supplementary text.

### Pathway visualization

To assist in the analysis of a large number of pathways designed using the modified optStoic approach, each pathway and reaction are represented as a Pathway Class object and Reaction Class object, respectively. A directed bipartite graph for each pathway is generated and rendered as SVG, PNG or JPEG format using the Graphviz software accessed through the Graphviz Python Package. The Python codes for pathway visualization are included in the *optstoic-python* package described in the previous section.

## Supplementary information

Supplementary Information

Supplementary Dataset

## Data Availability

All data generated or analyzed during this study are included in this published article.
