# Supplementary material for: Pareto Optimality Explanation of the Glycolytic Alternatives in Nature
Source: Sci Rep. 2019 Feb 22;9:2633. doi: 10.1038/s41598-019-38836-9 (PMC6384925; doi:10.1038/s41598-019-38836-9)
Supplement: Supplementary file 1 — Supplementary Information [file 41598_2019_38836_MOESM1_ESM.pdf]

Supplementary Information for

Pareto Optimality Explanation of the Glycolytic Alternatives in Nature

Chiam Yu Ng, Lin Wang, Anupam Chowdhury, and Costas D. Maranas

Corresponding author: Costas D. Maranas
Email: costas@psu.edu

This PDF file includes:

Supplementary Information Text
Figs. S1 to S8
Tables S1 to S2
References for SI reference citations

Other supplementary materials for this manuscript include the following:

Supplementary Data Files S1 to S6

Supplementary Information Text

Integer cut constraints and the runtime reduction of optStoic.

Integer cut constraints are introduced in the optStoic formulation to exhaustively identify alternate optimal pathways that satisfy the design equation. Herein, we define k as the number of iteration for running optStoic algorithm ($k \in \{1, 2, \dots, \kappa\}$).

The formulation of optStoic in the method section (see main text) was reformulated as a combination of linear relations by introducing two non-negative real number (or integer) variables for each v_j as followed:

$$v_j = v_j^f - v_j^r, \qquad \forall j \in \boldsymbol{J}$$

where $v_j \in \mathbb{Z}, v_j^f \in \mathbb{Z}_{\geq 0}$ and $v_j^r \in \mathbb{Z}_{\geq 0}$

or $\left(v_j \in \mathbb{R}, v_j^f \in \mathbb{R}_{\geq 0} \text{ and } v_j^r \in \mathbb{R}_{\geq 0}\right)$

$$|v_j| = v_j^f + v_j^r$$

To this end, binary variables y_j^f and y_j^r are defined as followed:

$$y_j^f = \begin{cases} 1, \text{ if reaction } j \text{ carries non-zero flux in the forward direction } (v_j^f > 0) \\ 0, \text{ otherwise} \end{cases} \qquad (1)$$

$$y_j^r = \begin{cases} 1, \text{ if reaction } j \text{ carries non-zero flux in the reverse direction } (v_j^r > 0) \\ 0, \text{ otherwise} \end{cases} \qquad (2)$$

Likewise, $y_j^{f^k}$ and $y_j^{r^k}$ are binary variables associated with the solution from the k-th iteration. At iteration $k = \kappa$, the following constraints are added to the modified optStoic formulation:

$$\sum_{j \in \boldsymbol{J} \mid y_j^{f^k} + y_j^{r^k} = 1} (1 - y_j^f - y_j^r) + \sum_{j \in \boldsymbol{J} \mid y_j^{f^k} + y_j^{r^k} = 0} (y_j^f + y_j^r) \geq 1 \qquad k = 1, 2, \dots, \kappa - 1 \qquad (3)$$

$$y_j^f + y_j^r \leq 1 \qquad (4)$$

$$y_j^f \varepsilon \leq v_j^f \leq y_j^f M \qquad (5)$$

$$y_j^r \varepsilon \leq v_j^r \leq y_j^r M \qquad (6)$$

Constraint 3 is the integer cut constraint that ensures that at least one of reaction j that was identified in the previous iteration k is inactive in the current iteration. Constraint 4 enforces that only one of the binary variables (corresponding to the flux directions) for each reaction j is active. Finally, constraints 5 and 6 restrict the flux (in forward or reverse direction) to be strictly positive whenever the corresponding binary variable is active. The parameter ε is a user-defined small positive real number. The MILP problems were solved using the CPLEX v.12.6.1 solver accessed through the GAMS (v24.4.1) modeling system and Gurobi Optimizer v6.5.1 using Python 2.7.

The runtime of the modified optStoic algorithm depends on the size of the search space (i.e. database size). Therefore, blocked reactions (i.e., reactions incapable of carrying flux)

were identified first upon imposing the bounds on exchange fluxes (see constraint 4 in main text) and excluded from the search space before running the algorithm. We also observed that the runtime of optStoic significantly increases when more integer cuts are added. This is caused by a large number of integer variables introduced in the second term of constraint 3 at each iteration k:

$$\sum_{j \in \boldsymbol{J} \,|\, y_j^{f^k} + y_j^{r^k} \neq 1} (y_j^f + y_j^r)$$

Upon removal of the blocked reactions, there are still over 3,000 reactions exist in the set $\{j \in \boldsymbol{J} \,|\, y_j^{f^k} + y_j^{r^k} = 0\}$. To solve this issue, we imposed an additional constraint on the objective function as followed:

$$\sum_{j \in \boldsymbol{J} \setminus \boldsymbol{J_{exchange}}} |v_j| = z^*$$

and used the following integer cut to prevent the same pathway from being identified for the same objective value z^*.

$$\sum_{j \in \boldsymbol{J} \,|\, y_j^{f^k} + y_j^{r^k} = 1} (1 - y_j^f - y_j^r) \geq 1, \qquad \forall\, k = 1, 2, 3, \ldots$$

We run the modified optStoic algorithm in parallel for each fixed objective value z^* to further reduce the total runtime.

Assessing the thermodynamic feasibility of a pathway.

The thermodynamic feasibility of each pathway under physiological concentration ranges are assessed using the max-min driving force (MDF) formulation (1).

Step 1: The $\Delta_r G'^{\circ}$for each reaction involved in a pathway ($j \in \boldsymbol{J_{path}}$) is estimated using the Component Contribution method (2) at pH 7, 25°C and ionic strength of 0.1 M (3, 4).

Step 2: The MDF problem is solved for each pathway, which minimizes the maximum $\Delta_r G_j'$ of a pathway by optimizing over the concentrations of all metabolites in the pathway. The optimization formulation is given by:

$$\max_{c_i} \quad \min_j \{-\Delta_r G_j'\} \qquad (MDF) \tag{7}$$

$$subject\ to\ \ \Delta_r G_j' = \Delta_r G_j'^{\circ} + RT \sum_{i \in \boldsymbol{I_{path}}} S_{ij}^T \ln \mathrm{c}_i\,, \qquad \forall\, j \in \boldsymbol{J_{path}} \tag{8}$$

$$\ln c_i^{min} \leq \ln \mathrm{c}_i \leq \ln c_i^{max}\,, \qquad \forall\, i \in \boldsymbol{I_{path}} \tag{9}$$

$$\ln r_p^{\min} \leq \ln r_p \leq \ln r_p^{\max}\,, \quad \forall\, p \in \boldsymbol{P} \tag{10}$$

where $\boldsymbol{I_{path}}$ is the set of all metabolites and $\boldsymbol{J_{path}}$ is the set of all reactions in a pathway, c_i is the concentration of metabolite i, R is the gas constant, T is the temperature, r_p is the concentration ratio for an ordered pair of metabolites p (e.g., $p = (ATP, ADP), r_{(ATP,ADP)} = c_{ATP}/c_{ADP}$), and $\boldsymbol{P}$ is a set of metabolite pairs (e.g., $\boldsymbol{P} \in \{(ATP, ADP), (NADPH, NADP^+), (NADH, NAD^+)\}$). Note that the S matrix here refers to the stoichiometric matrix of the pathway with $S \in \mathbb{R}^{|\boldsymbol{I_{path}}| \times |\boldsymbol{J_{path}}|}$.

Constraint 8 relates the Gibbs free energy of reaction ($\Delta_r G_j'$) with the standard Gibbs free energy of reaction ($\Delta_r G'^{\circ}{}_j$) and the mass action ratio. The concentrations of all metabolites are allowed to vary between 1 μM (c_i^{min}) and 100 mM (c_i^{max}) in constraint 9. Concentration ratios of common cofactor pairs (e.g., NADPH/$NADP^+$, NADH/NAD^+ and ATP/ADP) play an important role in a cell as they determine the driving force of a large number of biosynthesis reactions (5). The concentration ratios of energy and redox cofactors are therefore allowed to vary within the maximum and minimum values found in the literature (6-8) and the Bionumbers database (9) in constraint 10. Constraint 9 is optional depending on the case study. Herein, we assumed that the designed pathway operates at steady-state and within a single compartment of a cell at a temperature (T) of $25°C$, an ionic strength of 0.1 M and pH 7.0. The pathway with a positive objective function (MDF) indicates that it is thermodynamically infeasible within the given physiological concentration (and ratio) ranges is omitted from the subsequent step. Importantly, the objective function of the enzyme cost minimization problem is convex only when all $\Delta_r G_j' < 0$ in a pathway. The MDF problem is solved using Gurobi Optimizer v6.5.1 solver and Python script modified from the Component Contribution Python package (2).

We have previously found that when imposing the metabolite concentration ranges (constraint 9) strictly to experimentally measured metabolite levels, the MDF formulation is often over-constrained and may become infeasible due to several factors. They include: (i) measurement errors of the absolute intracellular metabolite concentrations, (ii) estimation errors of Gibbs free energy from group-contribution based approaches, (iii) the MDF formulation assumes that metabolite concentrations are homogenous (i.e., compartmentalization of metabolites and potential occurrence of substrate-channeling are ignored), and (iv) MDF analysis is performed with a pathway and not on the entire metabolic network. In addition, when we added the uncertainties in $\Delta_r G_j'^{\circ}$ to the MDF formulation (i.e., allowing the $\Delta_r G_j'^{\circ}$ of each reaction to vary between a range given by $\Delta_r G_j'^{\circ} \pm \mathrm{SE_j}$ in constraint 8), we found that MDF analysis will identify the more optimistic solution given the degree of freedom to have a more negative $\Delta_r G_j'^{\circ}$. Since, we are comparing between pathways, the relative contribution of the errors of $\Delta_r G_j'^{\circ}$ estimation has a lesser contribution towards the overall analysis and would not affect our conclusions. Therefore, MDF analysis of the pathways is studied within a larger physiological metabolite concentration ranges, and the errors of Gibbs free energy are not considered in the study.

Minimization of protein cost.

The minimal enzyme demand in units of mg protein/mmol glucose/h for each one of the thermodynamically feasible pathways is then estimated based on the enzyme cost minimization (ECM) method (10, 11). The formulation is as followed:

$$minimize \quad \mathrm{PC} = \frac{1}{v_{EX\_glc}} \sum_j M_{E,j} \lambda_{E,j} \qquad (ECM) \qquad (11)$$

$$subject\ to\ \lambda_{E,j} = \frac{v_j}{k^{+}_{cat,j}} \left(1 - \exp\left(\frac{\Delta_r G'_j}{RT}\right)\right)^{-1} \left(1 + \prod_{i \in \boldsymbol{I}_{\mathbf{re,j}}} \left(\frac{K_{M,ij}}{c_i}\right)^{q^{+}_{ij}}\right), \forall j \in \boldsymbol{J_{path}} \quad (12)$$

$$\Delta_r G'_j = \Delta_r G'^{\circ}_j + RT \ln \frac{\prod_{i \in \boldsymbol{I}_{\mathbf{pr,j}}} c_i^{q^{-}_{ij}}}{\prod_{i \in \boldsymbol{I}_{\mathbf{re,j}}} c_i^{q^{+}_{ij}}}, \qquad \forall j \in \boldsymbol{J_{path}} \quad (13)$$

$$\Delta_r G'_j < 0, \qquad \forall j \in \boldsymbol{J_{path}} \quad (14)$$

$$c_i^{min} \le \mathrm{c}_i \le c_i^{max}, \qquad \forall i \in \boldsymbol{I_{path}} \quad (15)$$

$$r_p^{\min} \le r_p \le r_p^{\max}, \qquad \forall p \in \boldsymbol{P} \quad (16)$$

where $M_{E,j}$ is the molecular weight of enzyme per active site for reaction j, v_{EX_glc} is the glucose uptake flux (mmol Glucose/h), $\lambda_{E,j}$ is the enzyme level for reaction j, v_j is the flux through reaction j, $k^{+}_{cat,j}$is the turnover number of the reaction in the forward direction, $\boldsymbol{I}_{\mathbf{re,j}}$ is the set of reactants in reaction j, $\boldsymbol{I}_{\mathbf{pr,j}}$ is the set of products in reaction j, the set of all metabolites in the pathway $\boldsymbol{I_{path}}$ is the union of $I_{\mathrm{re,j}}$ and $I_{\mathrm{pr,j}}$, $K_{M,ij}$ is the Michaelis-Menten constant of the enzyme for reaction j towards metabolite i, q^{+}_{ij} and q^{-}_{ij} is the stoichiometric coefficient of metabolite i in reaction j. $q^{+}_{ij} > 0$ if metabolite i is a reactant in reaction j and $q^{+}_{ij} = 0$ otherwise, whereas $q^{-}_{ij} > 0$ if metabolite i is a product in reaction j and $q^{-}_{ij} = 0$ otherwise. Note that in the preprocessing step, all the reactions are re-arranged such that flux v_j through each of them is strictly positive.

The objective function (equation 11) involves the minimization of the sum of the enzymatic cost (µg Protein/ mmol Glucose/ h) for each reaction in the pathway normalized by the glucose uptake rate. Constraint 12 defines the enzyme level for a reaction j as a function derived from the reversible Michaelis-Menten kinetic equation (10). Constraint 13 is equivalent to constraint 8 recasted using concentrations. Constraint 14 ensures that all reactions have a negative change in free energy and prevents division by zero in equation 12. Constraints 15 and 16 impose the bounds on the concentration ranges and concentration ratio ranges. The above formulation can be simplified by substituting the concentration variable c_i with logarithmic concentrations $x_i = \ln C_i$ and thus converting the product term into a summation.

According to Flamholz *et al.* (10), the enzyme cost minimization (ECM) formulation can be rewritten by substituting $x_i = \ln c_i$ as followed:

$$minimize\ \ \mathrm{PC} = \frac{1}{v_{EX\_glc}} \sum_j M_{E,j} \lambda_{E,j} \qquad (ECM) \quad (17)$$

$$subject\ to\ \ \lambda_{E,j} = \frac{v_j}{k_{cat,j}^{+}} \frac{1 + \exp\left(\sum_{i\in \boldsymbol{I}_{\mathbf{re,j}}} q_{ij}^{+}(\ln K_{M,ij} - x_i)\right)}{1 - \exp(\sum_{i\in \boldsymbol{I_{path}}} S_{ij}^{T} x_i + \frac{\Delta_r G_j'^{0}}{RT})}, \forall\, j \in \boldsymbol{J_{path}} \tag{18}$$

$$\Delta_r G_j' = \Delta_r G_j'^{\circ} + R \cdot T \cdot \sum_{i\in \boldsymbol{I_{path}}} S_{ij}^{T}\, x_i, \qquad \forall\, j \in \boldsymbol{J_{path}} \tag{19}$$

$$\Delta_r G_j' \leq 0 - \varepsilon, \qquad \forall\, j \in \boldsymbol{J_{path}} \tag{20}$$

$$\ln c_i^{min} \leq\ x_i \leq \ln c_i^{max}, \qquad \forall\, i \in \boldsymbol{I_{path}} \tag{21}$$

$$\ln r_p^{\min}\ \leq \ln r_p \leq \ln r_p^{\max}, \qquad \forall\, p \in \boldsymbol{P} \tag{22}$$

Using log-concentration simplifies the formulation as the product term in equations 12 and 13 can be replaced by the summation term in equations 18 and 19, respectively. Note that we have set ε to a very small number (i.e., 1e^{-6}) to ensure that the denominator of $\lambda_{e,j}$ does not become zero. We increase ε stepwise by 10-fold up to 0.1 if the optimization failed to converge at a lower ε value. If the optimization still fails to terminate successfully at $\varepsilon = 0.1$, we exclude the pathway from the final solution.

The optimal concentrations of metabolites obtained from the MDF problem are used as the initial condition for the ECM problem, which is then solved using the sequential least squares quadratic programming method (Python SciPy package). Due to the lack of experimentally measured kinetic parameters, we assumed generic values ($M_E = 40\ kDa, k_{cat} = 79\ s^{-1}\ and\ K_M = 200\ \mu M$) (12) for all kinetic parameters as was carried out in the original study (10). This implies that all enzymes were treated as equally fast in every pathway. The allowable metabolite concentration ranges are identical to that of the MDF analysis.

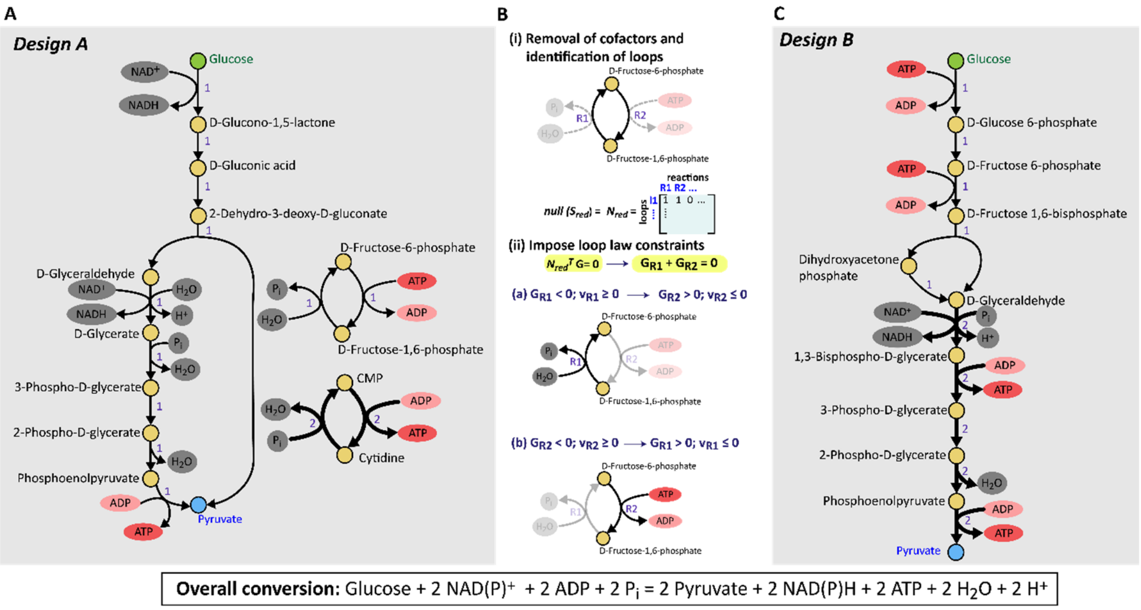

Fig. S1. Pathways shown here perform the overall conversion defined below the panels. (A) Design A is a pathway with disjoint subnetworks that generate cofactors such as ATP. This design is obtained using the previous optStoic formulation. (B) The $\boldsymbol{S_{int}}$ matrix, which contains only internal reactions, was processed by removing rows containing cofactors. The basis of the null space of the resulting $\boldsymbol{S_{red}}$ matrix is then obtained ($\boldsymbol{null(S_{red})} = \boldsymbol{N_{red}}$). Each row of the $\boldsymbol{N_{red}}$ matrix is an internal cycle that results in no net non-cofactor metabolite production. The loop law is imposed as $\boldsymbol{N_{red}^{T}G} = \mathbf{0}$, which implies that flux could traverse only through one of the directions in a loop. Two cases are shown here for the loop involving reaction R1 (D-Fructose-1,6-phosphate + H_2O → D-Fructose-6-phosphate + P_i) and R2 (D-Fructose-6-phosphate + ATP → D-Fructose-1,6-phosphate + ADP). In case (ii) (a), when reaction R1 is active ($\boldsymbol{v_{R1}} > \mathbf{0}$), then reaction R2 can carry only zero flux or flux in the same direction with R1. (C) After adding the loop law constraints, we found that ATP and redox generation occurs only on the main carbon transfer pathway.

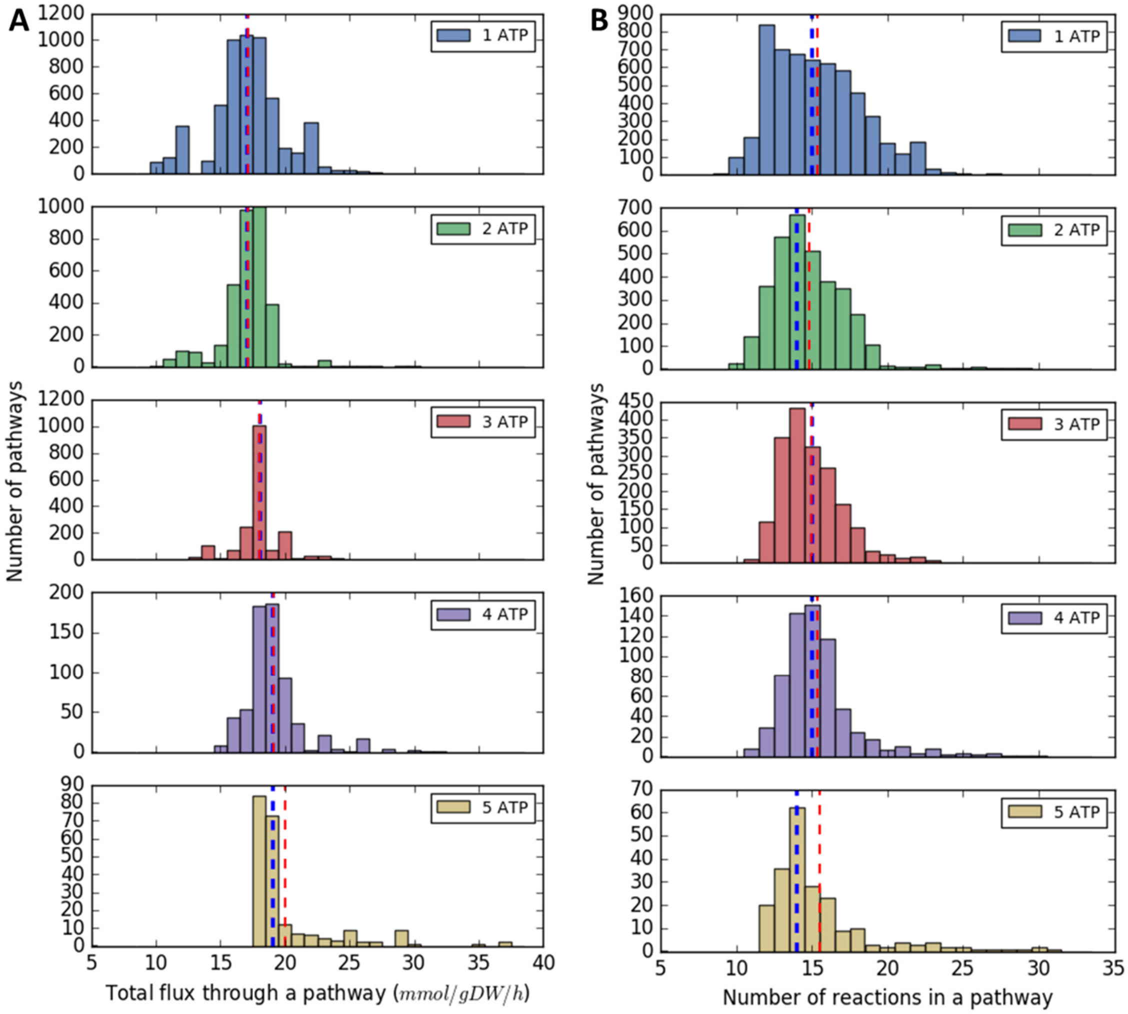

Fig. S2. The distribution of the glycolytic pathway alternatives based on (A) total flux through a pathway, and (B) the number of reactions in a pathway. Note that the total flux through a pathway and the number of reactions are calculated without accounting for the exchange reactions. The colors represent the ATP yield per glucose (mol ATP/mol glucose) generated by a pathway at a fixed glucose uptake flux. Red dashed lines indicate the mean values, whereas blue dashed lines denote the median values.

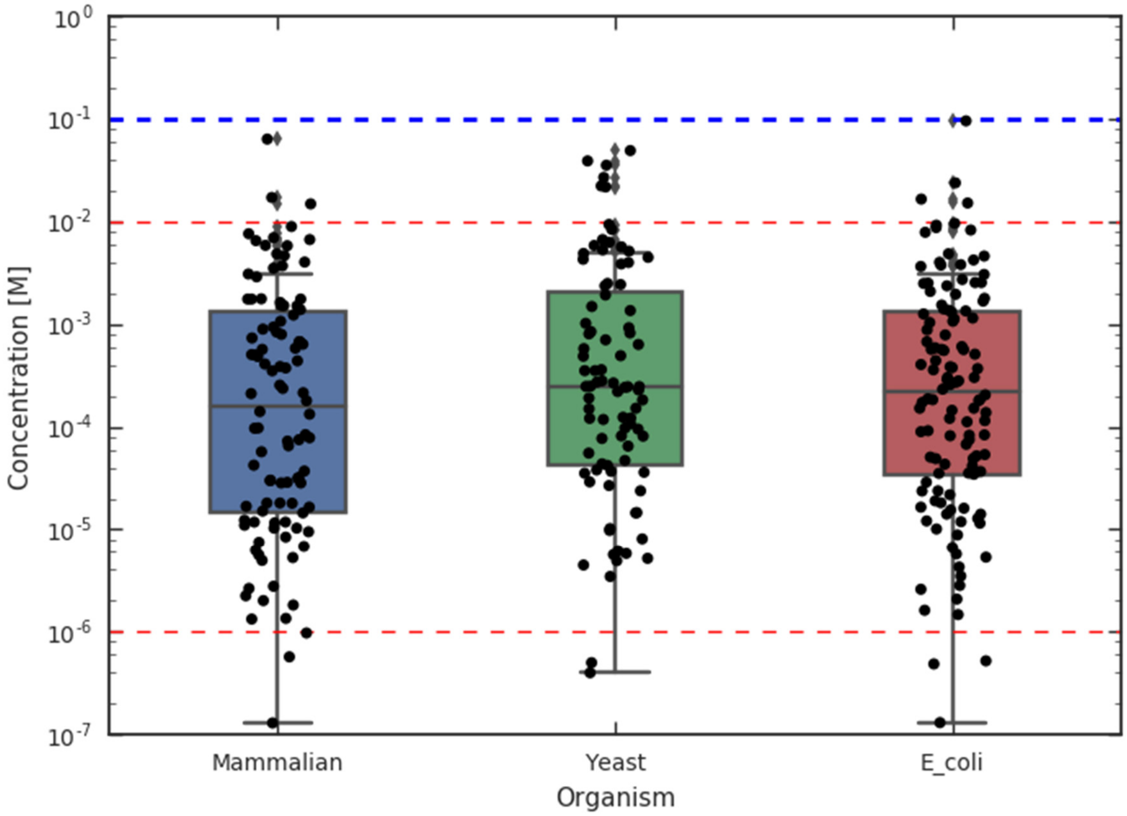

Fig. S3. Distribution of absolute metabolite concentrations across different organisms (8). The fraction of metabolites that are within 1 µM and 100 mM are 97.1%, 97.7% and 97.5% for mammalian cells, yeast and *E. coli*, respectively. The fraction of metabolites that fall within 1 µM and 10 mM are 94.1%, 90.9% and 94.2% for mammalian cells, yeast and *E. coli*, respectively.

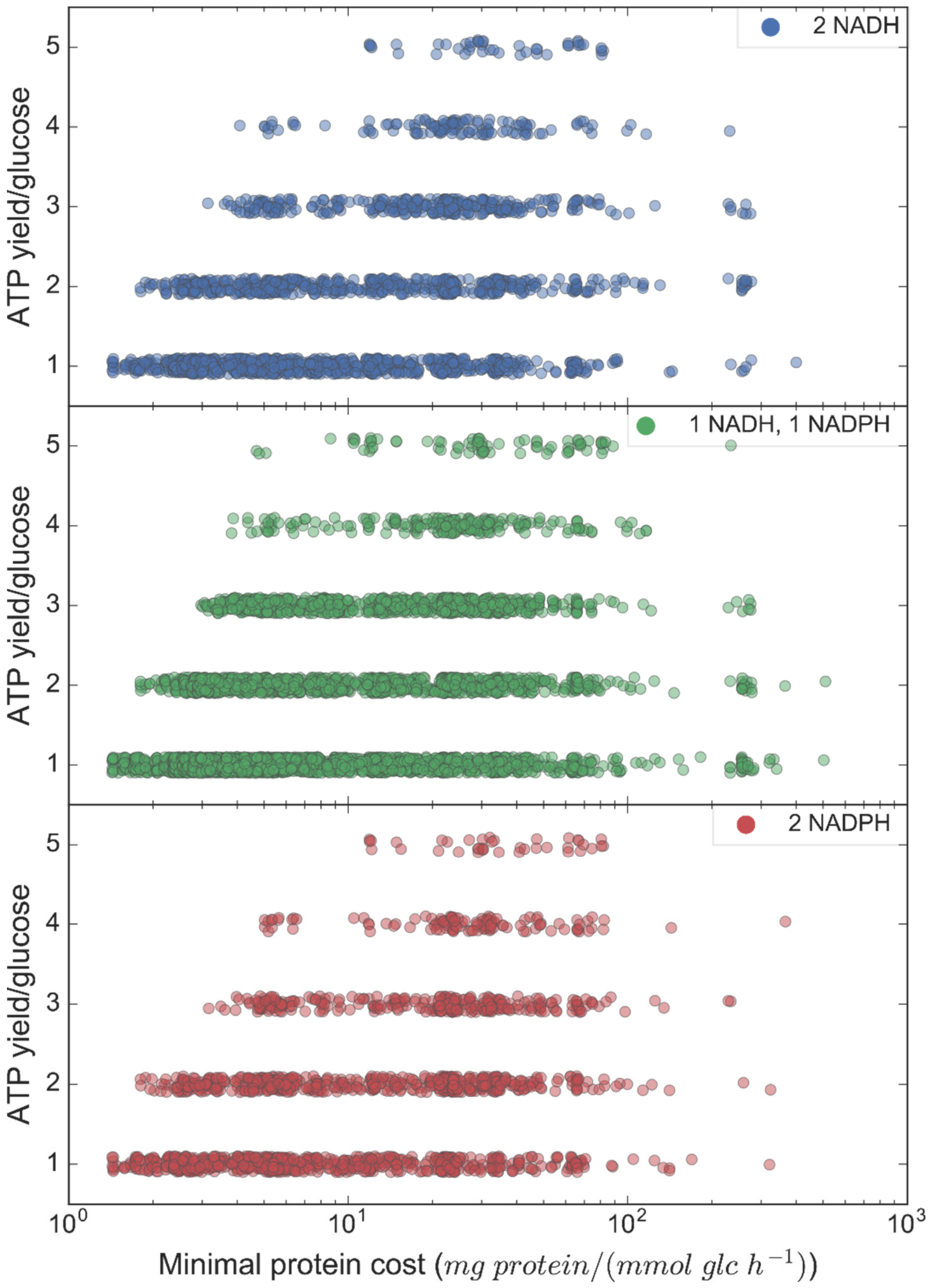

Fig. S4. The ATP yield versus minimal protein cost scatter plot. Note that the Y-axis is categorical. Jittering effect was applied to the plot to show the distribution more clearly. Pathways are color-coded based on the type of redox cofactors produced: (Blue) 2 NADH, (Green) 1 NADH and 1 NADPH, and (Red) 2 NADPH.

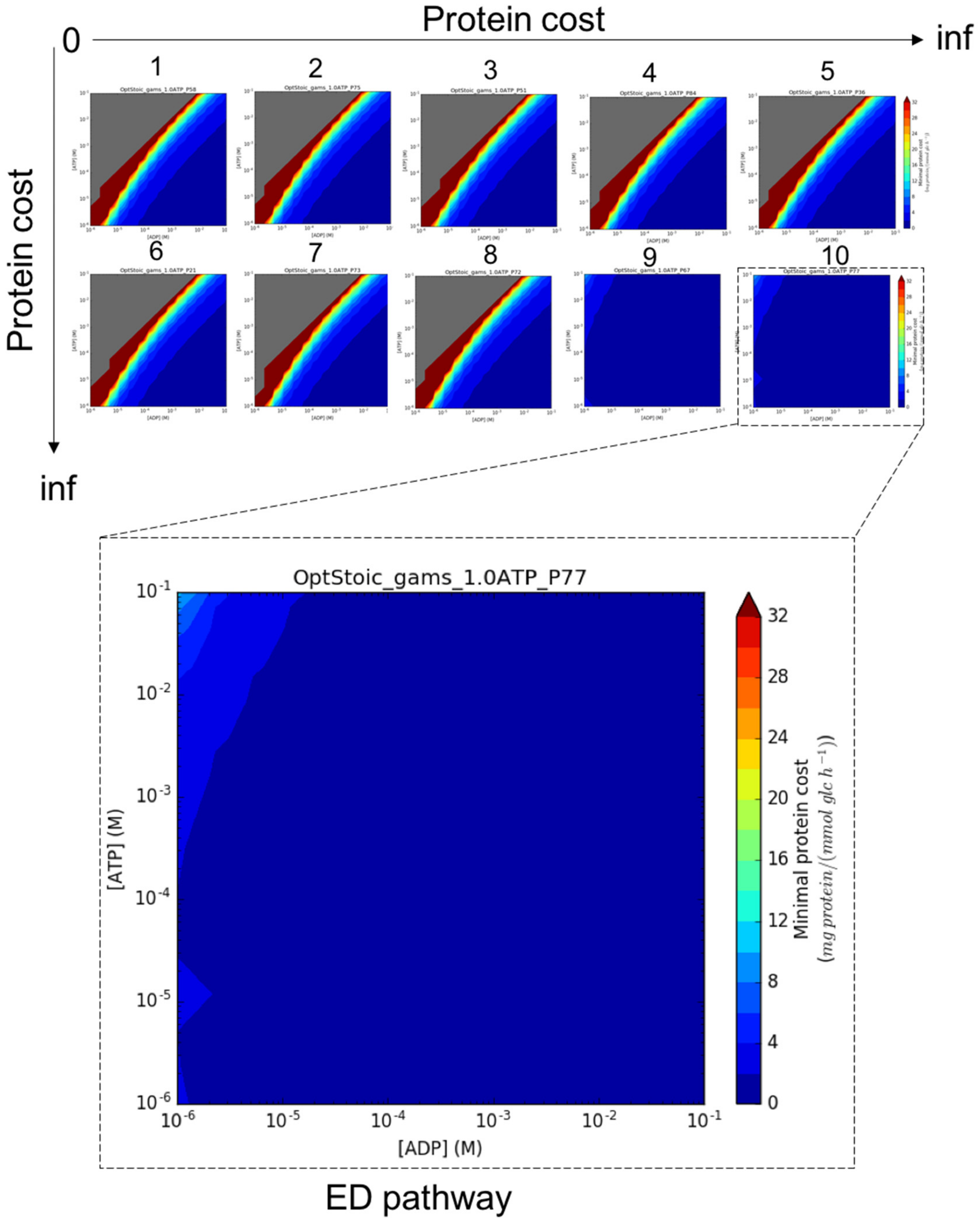

Fig. S5. Robustness analysis of the effect of ATP and ADP concentrations on the top ten 1-ATP generating pathways. Note that the legends for the x and y-axes are the same for all the ten pathways.

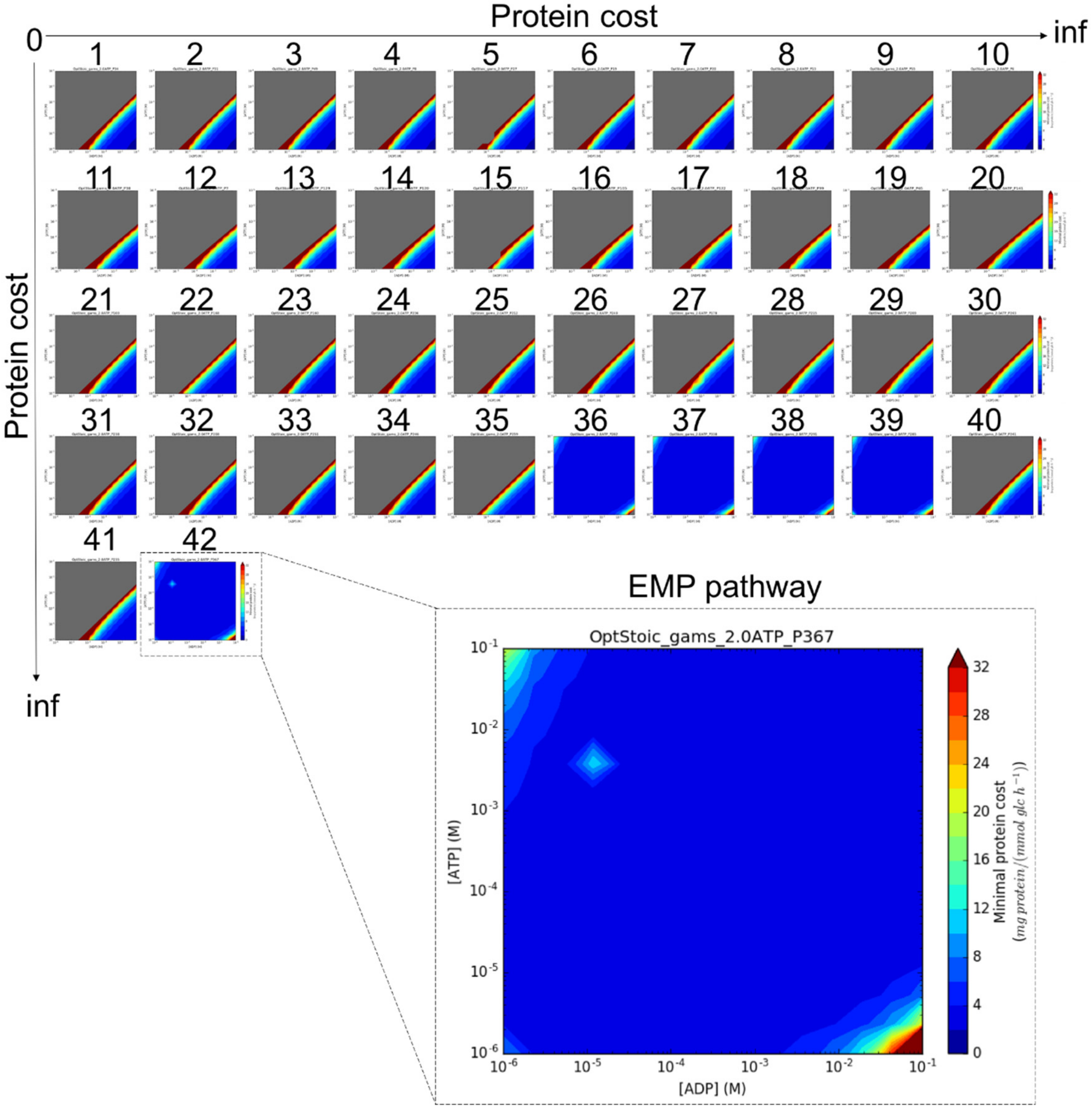

Fig. S6. Robustness analysis of the effect of ATP and ADP concentrations on the top forty-two 2-ATP generating pathways. Note that the legends for the x and y-axes are the same for all the 42 pathways. In addition, the ranges of the axes and heat map scales are the same for each panel.

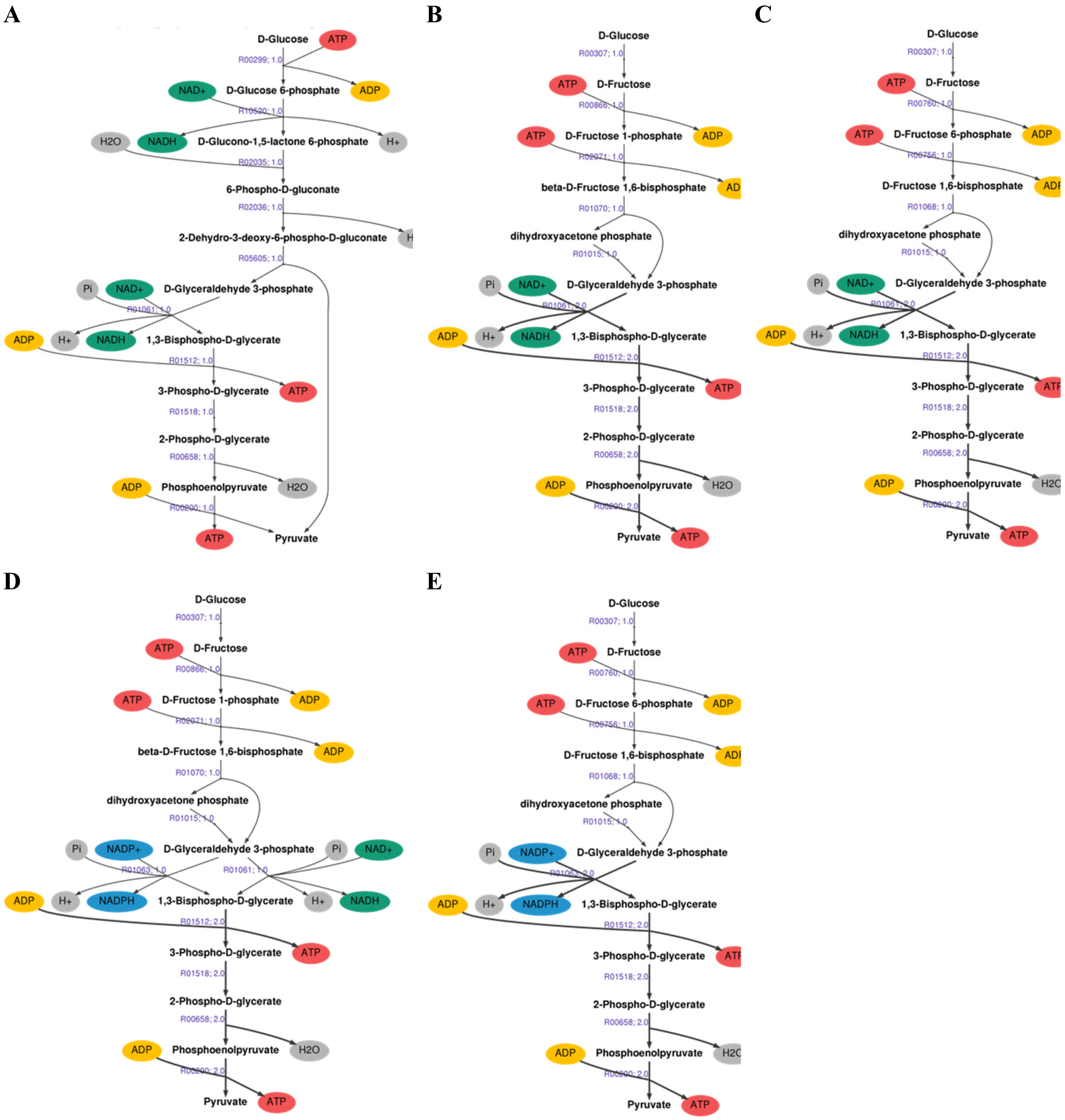

Fig. S7. Robust 1-ATP and 2-ATP generating glycolytic pathways within a broad range of ATP/ADP ratio. The pathway diagrams were generated using the pathway visualization tool described in the Method section. Pathway (A) is the 9th 1-ATP generating pathways ranked by protein cost in Fig. S5. Pathways (B), (C), (D) and (E) are 36th to 39th 2-ATP generating pathways ranked by protein cost in Fig. S6.

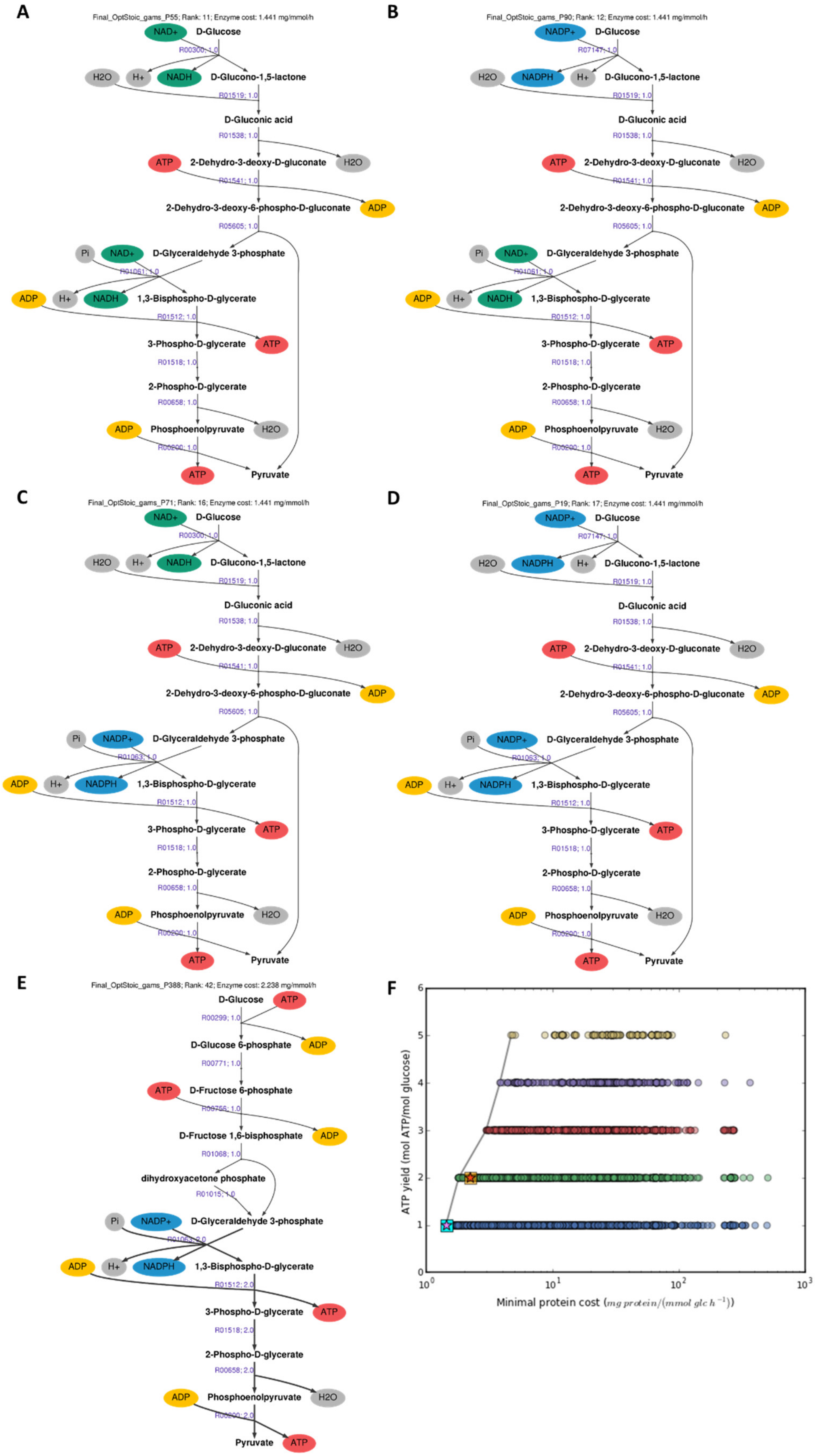
A
Final_OptStoic_gams_P55; Rank: 11; Enzyme cost: 1.441 mg/mmol/h
B
Final_OptStoic_gams_P90; Rank: 12; Enzyme cost: 1.441 mg/mmol/h
C
Final_OptStoic_gams_P71; Rank: 16; Enzyme cost: 1.441 mg/mmol/h
D
Final_OptStoic_gams_P19; Rank: 17; Enzyme cost: 1.441 mg/mmol/h
E
Final_OptStoic_gams_P388; Rank: 42; Enzyme cost: 2.238 mg/mmol/h
D-Glucose
D-Glucono-1,5-lactone
D-Gluconic acid
2-Dehydro-3-deoxy-D-gluconate
2-Dehydro-3-deoxy-6-phospho-D-gluconate
D-Glyceraldehyde 3-phosphate
1,3-Bisphospho-D-glycerate
3-Phospho-D-glycerate
2-Phospho-D-glycerate
Phosphoenolpyruvate
Pyruvate
D-Glucose 6-phosphate
D-Fructose 6-phosphate
D-Fructose 1,6-bisphosphate
dihydroxyacetone phosphate
NAD+
NADH
NADP+
NADPH
ATP
ADP
H2O
H+
Pi
F
ATP yield (mol ATP/mol glucose)
Minimal protein cost (mg protein/(mmol glc h⁻¹))

Fig. S8. (A-D) All four variants of the semi-phosphorylative ED pathway and (E) the NADPH-dependent EMP pathway described in the text. The pathway diagrams were generated using the pathway visualization tool described in the Method section. (F) The tradeoff plot of the minimal protein cost and the ATP yield of all glycolytic pathway variants as shown in Figure 3 (B). In addition to the ED (pink star) and the EMP pathways (red star), the semi-phosphorylative ED pathway variants (A-D) are represented as light-blue squares, whereas the NADPH-dependent EMP pathway is shown in the yellow square.

Table S1. Cofactors that were removed from the S matrix when generating the internal stoichiometric matrix ($\boldsymbol{S}^{*}$).

KEGG ID	Description	KEGG ID	Description
C00001	H2O	C00112	CDP
C00002	ATP	C00131	dATP
C00003	NAD+	C00138	Reduced ferredoxin
C00004	NADH	C00139	Oxidized ferredoxin
C00005	NADPH	C00144	GMP
C00006	NADP+	C00206	dADP
C00007	Oxygen	C00286	dGTP
C00008	ADP	C00360	dAMP
C00009	Orthophosphate	C00361	dGDP
C00010	CoA	C00362	dGMP
C00011	CO2	C00363	dTDP
C00013	Diphosphate	C00364	dTMP
C00015	UDP	C00365	dUMP
C00016	FAD	C00390	Ubiquinol
C00020	AMP	C00399	Ubiquinone
C00035	GDP	C00458	dCTP
C00044	GTP	C00459	dTTP
C00055	CMP	C00460	dUTP
C00063	CTP	C01352	FADH2
C00075	UTP		
C00080	H+		
C00081	ITP		
C00104	IDP		
C00105	UMP		

Table S2A. The number of pathways that are thermodynamically feasible (MDF < 0) at physiological concentration ranges and ratio.

Conditions	# of 1-ATP Pathways	# of 2-ATP Pathways	# of 3-ATP Pathways	# of 4-ATP Pathways	# of 5-ATP Pathways
optStoic	5,739	3,430	1,873	659	215
condition (i)	4,550	2,891	1,542	466	165
condition (ii)	2,549	1,099	281	4	0
condition (iii)	2,525	1,098	281	4	0
condition (iv)	1,824	778	173	2	0
condition (v)	538	105	0	0	0
condition (vi)	2,558	1,099	281	4	0

i. All metabolites are allowed to vary between 1 μM and 100 mM.
ii. Same with (i), except that ATP and ADP concentrations are bounded based on Park *et al.* (8) ($\text{i.e.}, 1.66\text{ mM} \le C_{ATP} \le 11.4\text{ mM};\ 0.429\text{ mM} \le C_{ADP} \le 0.715\text{ mM}$).
iii. Same with (ii), except that the concentration range of CO_2 was bounded based on Park *et al.* (8) (i.e., 50 μM $\le C_{CO_2} \le$ 10 mM).
iv. All metabolites are allowed to vary between 1 μM and 100 mM except CO_2. The range of CO_2 was obtained from Park *et al.* (8) (i.e., 50 μM $\le C_{CO_2} \le$ 10 mM). The ratio ranges for different cofactor pairs were imposed as followed: $0.2 \le \frac{C_{ATP}}{C_{ADP}} \le 20$, $0.2 \le \frac{C_{NADPH}}{C_{NADP}} \le 100$, $0.0005 \le \frac{C_{NADH}}{C_{NAD}} \le 0.5$ based on data collected from Bionumbers (9) and literature (6-8).
v. All metabolites other than CO_2 are allowed to vary between 1 μM and 10 mM. The range of CO_2 was obtained from Park *et al.* (8) (i.e., 50 μM $\le C_{CO_2} \le$ 10 mM). The ratio ranges for different cofactor pairs were imposed as followed: $0.2 \le \frac{C_{ATP}}{C_{ADP}} \le 20$, $0.2 \le \frac{C_{NADPH}}{C_{NADP}} \le 100$, $0.0005 \le \frac{C_{NADH}}{C_{NAD}} \le 0.5$.
vi. All metabolites are allowed to vary between 1 μM and 100 mM. The ratio ranges for different cofactor pairs were imposed as followed: $1 \le \frac{C_{ATP}}{C_{ADP}} \le 10{,}000$.

Table S2B. All the constraints used for the simulation of Table S2A.

**Conditions**	**(i)**	**(ii)**	**(iii)**	**(iv)**	**(v)**	**(vi)**
(A) $1\ \mu M \le C_i \le 100\ mM$ for all metabolite i	+	+	+	+		+
(B) $1.66\ mM \le C_{ATP} \le 11.4\ mM$; $0.429\ mM \le C_{ADP} \le 0.715\ mM$		+	+			
(C) $50\ \mu M \le C_{CO_2} \le 10\ mM$			+	+	+	
(D) $0.2 \le \frac{C_{ATP}}{C_{ADP}} \le 20$; $0.2 \le \frac{C_{NADPH}}{C_{NADP}} \le 100$; $0.0005 \le \frac{C_{NADH}}{C_{NAD}} \le 0.5$				+	+	
(E) $1\ \mu M \le C_i \le 10\ mM$, for all metabolite i					+	
(F) $1 \le \frac{C_{ATP}}{C_{ADP}} \le 10{,}000$						+

References

1. Noor E*, et al.* (2014) Pathway thermodynamics highlights kinetic obstacles in central metabolism. *PLoS computational biology* 10(2):e1003483.
2. Noor E, Haraldsdottir HS, Milo R, & Fleming RM (2013) Consistent estimation of Gibbs energy using component contributions. *PLoS computational biology* 9(7):e1003098.
3. Biemans-Oldehinkel E, Mahmood NA, & Poolman B (2006) A sensor for intracellular ionic strength. *Proceedings of the National Academy of Sciences of the United States of America* 103(28):10624-10629.
4. Storey KB (2004) *Functional Metabolism: Regulation and Adaptation* (John Wiley & Sons, Inc., Hoboken, NJ, USA) p 616.
5. Zhang J*, et al.* (2015) Determination of the Cytosolic NADPH/NADP Ratio in Saccharomyces cerevisiae using Shikimate Dehydrogenase as Sensor Reaction. *Scientific Reports* 5:12846.
6. Bennett BD*, et al.* (2009) Absolute metabolite concentrations and implied enzyme active site occupancy in Escherichia coli. *Nature chemical biology* 5(8):593-599.
7. Gerosa L*, et al.* (2015) Pseudo-transition Analysis Identifies the Key Regulators of Dynamic Metabolic Adaptations from Steady-State Data. *Cell Syst* 1(4):270-282.
8. Park JO*, et al.* (2016) Metabolite concentrations, fluxes and free energies imply efficient enzyme usage. *Nature chemical biology*.
9. Milo R, Jorgensen P, Moran U, Weber G, & Springer M (2010) BioNumbers--the database of key numbers in molecular and cell biology. *Nucleic acids research* 38(Database issue):D750-753.
10. Flamholz A, Noor E, Bar-Even A, Liebermeister W, & Milo R (2013) Glycolytic strategy as a tradeoff between energy yield and protein cost. *Proceedings of the National Academy of Sciences of the United States of America* 110(24):10039-10044.
11. Noor E*, et al.* (2016) The Protein Cost of Metabolic Fluxes: Prediction from Enzymatic Rate Laws and Cost Minimization. *PLoS computational biology* 12(11):e1005167.
12. Bar-Even A*, et al.* (2011) The moderately efficient enzyme: evolutionary and physicochemical trends shaping enzyme parameters. *Biochemistry* 50(21):4402-4410.
